# An investigation of the internal morphology of asbestos ferruginous bodies: constraining their role in the onset of malignant mesothelioma

**DOI:** 10.1186/s12989-023-00522-0

**Published:** 2023-05-08

**Authors:** Maya-Liliana Avramescu, Christian Potiszil, Tak Kunihiro, Kazunori Okabe, Eizo Nakamura

**Affiliations:** 1 The Pheasant Memorial Laboratory for Geochemistry and Cosmochemistry, Institute for Planetary Materials, Okayama University, Misasa, Tottori 682-0193 Japan; 2 Bell Land General Hospital, 500-3 Higashiyama, Sakai, Osaka 599-8247 Japan; 3 Advanced Science Research Center, Okayama University, Tsushima, Okayama 700-8530 Japan

**Keywords:** Asbestos fibre, Asbestos body, Malignant mesothelioma, Asbestos body internal morphology

## Abstract

**Background:**

Asbestos is a fibrous mineral that was widely used in the past. However, asbestos inhalation is associated with an aggressive type of cancer known as malignant mesothelioma (MM). After inhalation, an iron-rich coat forms around the asbestos fibres, together the coat and fibre are termed an “asbestos ferruginous body” (AFB). AFBs are the main features associated with asbestos-induced MM. Whilst several studies have investigated the external morphology of AFBs, none have characterised the internal morphology. Here, cross-sections of multiple AFBs from two smokers and two non-smokers are compared to investigate the effects of smoking on the onset and growth of AFBs. Morphological and chemical observations of AFBs were undertaken by transmission electron microscopy, energy dispersive x-ray spectroscopy and selected area diffraction.

**Results:**

The AFBs of all patients were composed of concentric layers of 2-line or 6-line ferrihydrite, with small spherical features being observed on the outside of the AFBs and within the cross-sections. The spherical components are of a similar size to Fe-rich inclusions found within macrophages from mice injected with asbestos fibres in a previous study. As such, the spherical components composing the AFBs may result from the deposition of Fe-rich inclusions during frustrated phagocytosis. The AFBs were also variable in terms of their Fe, P and Ca abundances, with some layers recording higher Fe concentrations (dense layers), whilst others lower Fe concentrations (porous layers). Furthermore, smokers were found to have smaller and overall denser AFBs than non-smokers.

**Conclusions:**

The AFBs of smokers and non-smokers show differences in their morphology, indicating they grew in lung environments that experienced disparate conditions. Both the asbestos fibres of smokers and non-smokers were likely subjected to frustrated phagocytosis and accreted mucopolysaccharides, resulting in Fe accumulation and AFB formation. However, smokers’ AFBs experienced a more uniform Fe-supply within the lung environment compared to non-smokers, likely due to Fe complexation from cigarette smoke, yielding denser, smaller and more Fe-rich AFBs. Moreover, the lack of any non-ferrihydrite Fe phases in the AFBs may indicate that the ferritin shell was intact, and that ROS may not be the main driver for the onset of MM.

**Supplementary Information:**

The online version contains supplementary material available at 10.1186/s12989-023-00522-0.

## Background

Asbestos is a fibrous silicate mineral widely used for its beneficial characteristics [1], which include incombustibility, heat resistance and flexibility. By the 1990s, asbestos was known to cause malignant mesothelioma (MM), an aggressive occupational cancer, leading to the widespread ban of asbestos [2]. Consequently, asbestos is now classified as a human lung carcinogen [3]. The cases of MM are expected to greatly increase as MM has a long latency period (15–50 years) [2, 4].

The asbestos fibres are inhaled into the lung environment where they are phagocytosed by the alveolar macrophages (AM). However, the AM fail to phagocytose the asbestos fibres due to the fibres’ long lengths, leading to “frustrated phagocytosis” [2, 4]. Following frustrated phagocytosis, the AM release pro-inflammatory cytokines, free radicals, and reactive oxygen species (ROS), while the asbestos fibres continue to persist in the lung environment [4–6]. The amosite (grunerite) (Fe^2^_7_^+^(Si_8_O_22_)(OH)_2_) and crocidolite (riebeckite) (Na_2_Fe^2^_3_^+^Fe^3^_2_^+^(Si_8_O_22_)(OH)_2_) fibres are known to persist in the lung environment [7–9] whereas chrysotile (Mg_3_(Si_2_O_5_)(OH)_4_) fibres are known to dissolve within the lung environment due to the dissolution of their brucite layer [7, 10, 11]. The AM also scavenge Fe from senescent red blood cells, storing the Fe in ferritin (an Fe transport protein), within the AM for Fe recycling [12].

While in the lung environment, the asbestos fibres accumulate Fe and ferritin containing ferrihydrite (Fe^3 +^ _10_O_14_(OH)_2_), together with acid mucopolysaccharides, lung surfactant proteins and phospholipids onto their surfaces [1, 6, 10, 13–18]. The accumulated products accrete as an Fe coat onto the asbestos fibre, resulting in the asbestos ferruginous body (AFB) [6, 10, 13, 19, 20]. While the Fe coat of the AFB has been suggested to protect against the irritant nature of the asbestos fibre, it is also thought to be involved in Fe-mediated ROS production [17, 20, 21].

Pascolo et al., 2016, 2016 [22, 23] investigated the occurrence of asbestos fibres and AFBs in tissue samples using X-ray fluorescence (XRF) to achieve better detection than conventional histochemical procedures. The studies also investigated Fe and Ca deposition on asbestos fibres. Pascolo et al. 2016 [22] reported that Fe and Ca homeostasis were disrupted by the asbestos fibres which led to the deposition of these elements onto the fibres. The Fe accumulated onto the asbestos fibres was in the form of a misfolded ferritin protein. Pascolo et al., 2016 [22] speculated that the misfolding of the ferritin was induced by both the process of its adsorption onto the fibre and the release of cytosolic Ca, eventually leading to loss of ferritin function, the release of Fe and ROS production.

Bardelli et al., 2017 [20] observed AFBs using XRF and scanning electron microscopy (SEM). Accordingly, a speculative model was proposed where the inner part of AFBs is richer in Fe than the outer part, due to the overloaded ferritin transforming into hemosiderin. However, it is difficult to distinguish between ferritin and hemosiderin. The study’s alternative scenario involved the release of Fe from the fibre, which then gradually dissipates throughout the AFB.

Di Giuseppe et al., 2019 [11] performed analysis only on the exterior of AFBs using transmission electron microscopy (TEM) and energy dispersive X-ray spectrometry (EDS). The study’s selected area diffraction (SAED) patterns identified other crystal materials than ferrihydrite, such as goethite (α-Fe^3+^O(OH)). The aforementioned observations were attributed to the degradation of the ferritin shell on the asbestos fibre, which led to the exposure of the ferrihydrite inside the ferritin. Di Giuseppe et al., 2019 [11] suggested that the exposed ferrihydrite either altered to goethite or it contributed to the release of Fe that induced ROS through the Fenton reaction.

Previous AFB studies reflect morphological descriptions and chemical compositions from only the exterior of the AFB [11, 20, 22, 23]. Thus, previous studies have not been able to investigate the relationships between asbestos fibres and variations present within the AFBs from their interior to their exterior. Therefore, the aim of this study was to observe the interior of the AFBs, by slicing them using a focused ion beam (FIB) and investigate the chemical and morphological variation within them. Here the internal structure of the AFBs will be observed by TEM and EDS in order to shed more light on AFB generation and evolution within the human lung environment. Furthermore, the current study will also investigate the potential effects of cigarette smoking (CS) on the formation of AFBs.

**Table 1:** Information regarding asbestos fibres and AFBs: external lengths, widths and type of the AFB fibres and bodies, and internal diameters of the fibres and bodies.

Patient	AFB	Fibre			Body		Fibre-1 Diameter		Fibre-2 Diameter		Fibre-3 Diameter		Body Diameter	
		length	width	Type	length	width	long	short	long	short	long	short	long	short
N1	1	26.67	0.37	Amosite	20.88	5.21 ± 1.29	0.86	0.32	-	-	-	-	6.22	6.22
	2	29.39	1.07	Amosite	24.59	10.00	0.97	0.56	-	-	-	-	8.43	7.39
	3	51.80	0.83	Amosite	16.94	6.73 ± 2.05	0.80	0.54	0.49	0.33	-	-	8.05	-
	4	71.10	1.71	Amosite	42.55	9.03	1.62	1.10	0.48	0.28	-	-	7.72	-
	Average	44.74 ± 20.87	1.00 ± 0.56		26.24 ± 11.31	7.74 ± 2.18	1.06 ± 0.38	0.63 ± 0.33	0.49 ± 0.01	0.31 ± 0.04	-	-	7.61 ± 0.97	6.81 ± 0.83
N2	1	15.89	0.42	Crocidolite	8.59	5.86	0.63	0.42	-	-	-	-	5.45	4.93
	2	19.90	0.42	Crocidolite	17.53	2.24 ± 0.79	0.42	0.35	-	-	-	-	3.11	3.09
	3	22.19	0.40	Amosite	6.40	3.51 ± 1.00	0.45	0.33	-	-	-	-	4.52	3.98
	4	20.28	0.39	Crocidolite	16.92	5.22 ± 0.4	0.49	0.13	-	-	-	-	5.54	5.21
	Average	19.57 ± 2.65	0.41 ± 0.02		12.36 ± 5.69	4.21 ± 1.65	0.50 ± 0.09	0.31 ± 0.12	-	-	-	-	4.66 ± 1.13	4.30 ± 0.96
S1	1	24.87	0.70	Amosite	18.30	2.81	0.92	0.32	-	-	-	-	2.56	2.01
	2	7.01	0.14	-	6.54	2.18 ± 1.7	0.27	0.06	-	-	-	-	3.29	3.20
	3	48.29	0.89	Amosite	20.22	3.69	0.88	0.26	-	-	-	-	4.51	3.00
	4	21.18	1.02	Amosite	5.32	4.15 ± 0.16	1.00	0.26	-	-	-	-	3.94	3.75
	Average	25.34 ± 17.13	0.69 ± 0.39		12.6 ± 7.75	3.21 ± 0.88	0.77 ± 0.34	0.22 ± 0.11	-	-	-	-	3.58 ± 0.84	2.99 ± 0.73
S2	1	9.92	0.32	Crocidolite	9.55	2.78	0.32	0.19	-	-	-	-	2.29	2.12
	2	22.91	0.19	-	10.57	2.96	-	-	-	-	-	-	2.69	2.59
	3	16.41	0.24	Crocidolite	13.25	2.10 ± 0.35	0.26	0.06	0.18	0.08	0.19	0.07	2.07	1.61
	Average	16.41 ± 6.5	0.25 ± 0.07		11.12 ± 1.91	2.61 ± 0.45	0.29 ± 0.04	0.13 ± 0.09					2.35 ± 0.31	2.11 ± 0.49

## Results

### External morphology

**Fig. 1:**
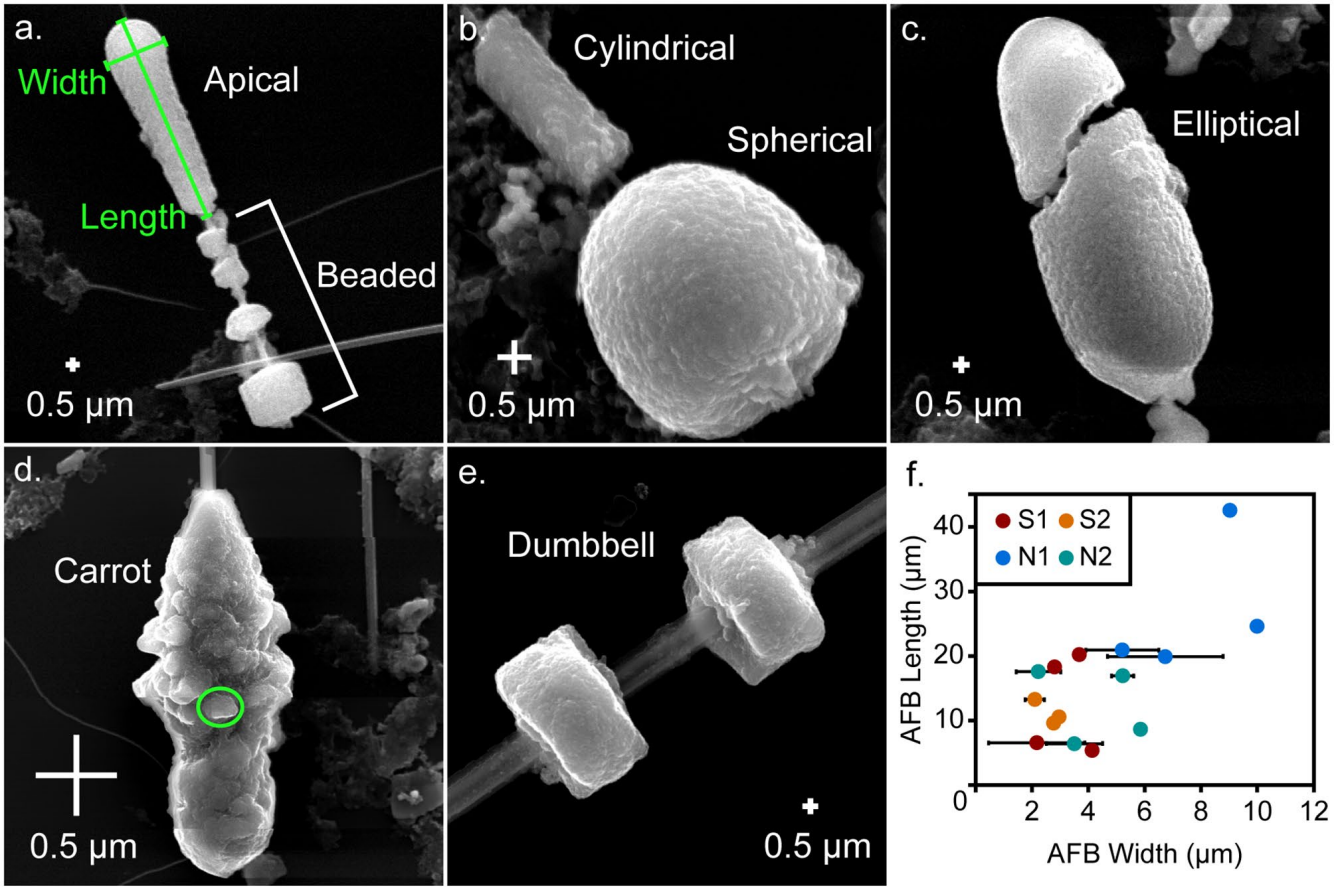
SEM images of the external morphology of different AFBs analysed by the current study: **a**. an apical form and beaded AFB from patient N1, **b**. cylindrical and spherical forms from an AFB from patient S1, **c**. an elliptical form from an AFB from patient N1, **d**. a carrot form from an AFB from patient N1, **e**. a dumbbell form from an AFB from patient S1 and **f**. a plot of average AFB form width against AFB length. A typical length and width measurement are shown in f., with the width and length being taken along the longest dimensions. The circle in d. highlights the sphere or elliptical-like components that form the AFB coats, with this particular component being 120 nm by 220 nm in diameter. N1 and N2 are non-smoking patient 1 and 2 and S1 and S2 are smoking patient 1 and 2, respectively

The external morphology of the AFBs was observed by SEM. The AFBs investigated here, were composed of fibres surrounded by a coat. The coat was not uniform across the entire fibre, being thick in places and almost absent in others (Fig. 1 and S1). The thicker areas could form large continuous regions or be segmented into various forms, giving a beaded appearance (Fig. 1a). Note that descriptions of the AFB forms were made using the nomenclature of a previous study [11]. Among the morphologies observed here, were spherical (Fig. 1b), cylindrical (Fig. 1b), elliptical (Fig. 1c), carrot-shaped (Fig. 1d), dumbbell-shaped (Fig. 1e) and apical (Fig. 1a) forms. The lengths of the AFBs and the widths of the AFB forms were measured (Fig. 1f and Table 1). While the lengths of the AFBs did not show any clear differences between the patients studied here, except that N1 recorded longer and thicker AFBs than the other patients did, the non-smoking patients recorded AFBs that were generally wider than the smoking patients.

Most of the selected AFBs featured small, spherical, or elliptical components (0.05–0.17 µm) (Fig. 1b, d) which had also adhered to the naked asbestos fibres. The internal portions of the AFBs (discussed later) also showed some characteristics consistent with the spheres observed on the outside of the AFBs, which suggests that the AFBs may have originally been composed of a substantial quantity of these spheres. The spheres appear to be randomly distributed but are more visible within the exterior portions of the AFB cross-sections. Further SEM images of the Fe-rich spheres and information concerning the AFBs selected here for TEM analysis, can be found in the Additional file 1 (Figure S1).

### Internal morphology

The internal morphology of the cross-sections of AFBs was observed by TEM dark field (DF) imaging (Fig. 2 and Additional File 2 with Figures S2-S16). The wavy white discolouration on the surface of the AFBs is a result of the bodies adhering to the biofilm of the TEM copper grid.

**Fig. 2:**
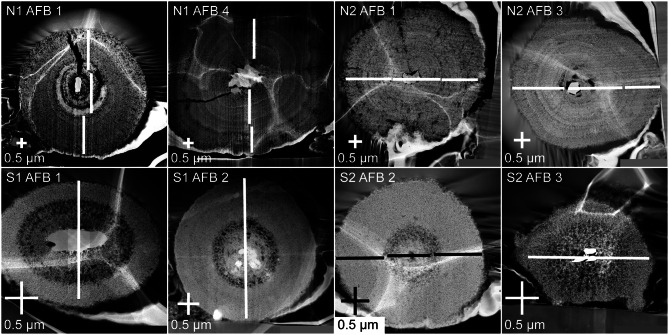
TEM dark-field images of cross-sectional views from AFBs of each patient. Two AFBs were selected from each patient; the patient and AFB number are indicated in the top left-hand corner of each panel. The white or black lines indicate the transects along which the EDS data was taken. Note that the wavy white discolouration on the surface of the AFBs is from the copper biofilm that was used to fix the AFBs in place. N1 and N2 are non-smoking patient 1 and 2 and S1 and S2 are smoking patient 1 and 2, respectively

The AFBs of all patients demonstrated concentric layers of different shades in their TEM DF images. The darker layers appeared darker due to the presence of small holes within the layers which are termed porous layers here. Meanwhile, some layers in a given AFB TEM DF image were brighter and without small holes which were termed dense layers. It was apparent that the AFBs of the non-smokers demonstrated a larger number of concentric layers than those of the smokers. Furthermore, the smokers AFBs tended to feature a thick, porous layer close to the fibre or immediately surrounding it.

**Fig. 3:**
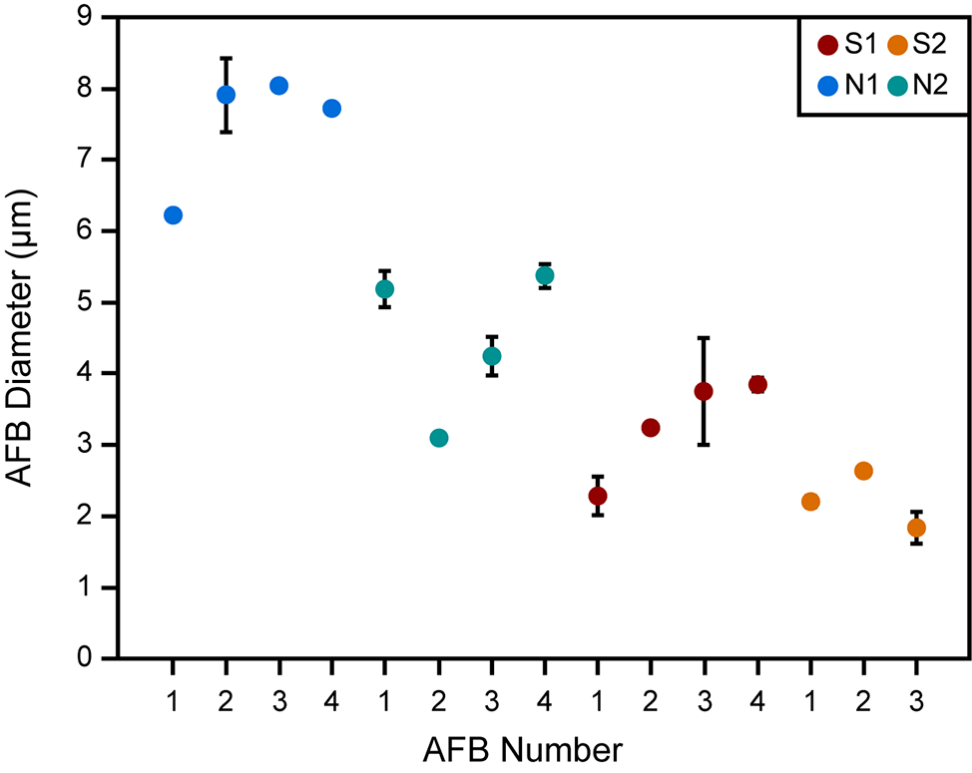
A representation of the diameters of AFBs from all patients analysed here. The spot represents the average value calculated from two measurements, one of the longest diameter and one of the shortest diameter, while the ends of the error bar indicate the highest and lowest diameter values. Note that for AFBs that were almost perfectly circular, error bars are not present. The values were measured using the TEM DF images shown in Fig. 2 and the Additional file 2 (Figures S2-S16). N1 and N2 are non-smoking patient 1 and 2 and S1 and S2 are smoking patient 1 and 2, respectively. AFB number refers to the number used to identify the AFB that is used throughout the manuscript

The longest and shortest diameters of the exposed cross-sections of the AFBs and their fibres were also measured (Table 1). As expected from the external AFB width data, the AFB diameters are generally larger for the non-smoking patients compared to the smoking patients (Fig. 3). In bodies such as AFB 2 of patient S1, white circles were a result of EDS damage.

**Table 2:** EDS elemental quantification (wt. %) of analysed AFBs: NS-AFB (AFBs of non-smoking patients N1 and N2), S-AFB (AFBs of smoking patients S1 and S2) and S-fibre (asbestos fibres of AFBs of non-smoking patient N2 and smoking patients S1 and S2)

		O	Mg	Al	Si	P	Ca	Mn	Fe
	Mean (n = 4)	28.8 ± 1.88	0.76 ± 0.25	1.49 ± 0.39	6.18 ± 2.97	1.73 ± 0.35	1.79 ± 0.38	0.11 ± 0.11	59.14 ± 4.82
N1-AFB	Min	25.49	0.19	0.4	1.37	0.94	0.91	0	34.06
	Max	38.67	1.63	3.58	22.41	2.69	3.70	0.45	67.96
	Mean (n = 4)	28.70 ± 3.04	0.73 ± 0.23	1.41 ± 0.4	6.37 ± 4.75	1.53 ± 0.34	1.58 ± 1.19	0.48 ± 0.16	59.20 ± 7.96
N2-AFB	Min	25.47	0.00	0.27	1.16	0.09	0.49	0.00	5.01
	Max	50.82	1.72	4.90	41.45	2.70	14.97	1.20	67.84
	Mean (n = 4)	27.08 ± 1.6	0.58 ± 0.36	1.03 ± 0.34	4.19 ± 3.51	1.76 ± 0.34	1.99 ± 0.59	0.08 ± 0.1	63.13 ± 5.45
S1-AFB	Min	25.36	0.17	0.03	1.48	0	0.14	0	31.14
	Max	40.19	2.83	3.61	26.07	2.25	3.93	0.65	67.75
	Mean (n = 3)	27.23 ± 1.28	0.64 ± 0.24	1.30 ± 0.53	4.16 ± 1.7	1.64 ± 0.31	1.50 ± 0.55	0.10 ± 0.08	63.43 ± 3.47
S2-AFB	Min	25.58	0.34	0.68	2.31	1.03	0.63	0	51.04
	Max	30.87	1.95	4.21	9.07	2.38	3.28	0.32	68.14
	Mean (n = 4)	41.68 ± 2.46	1.27 ± 0.54	0.09 ± 0.08	28.51 ± 3.95	0.06 ± 0.15	0.66 ± 0.79	0.10 ± 0.10	27.63 ± 5.74
N2-Fibre	Min	39.48	0.37	0.00	24.12	0.00	0.08	0.00	11.87
	Max	48.35	2.24	0.32	38.94	0.69	3.59	0.45	33.09
	Mean (n = 4)	37.57 ± 3.55	1.94 ± 0.8	0.2 ± 0.34	21.74 ± 6.07	0.26 ± 0.55	0.45 ± 0.6	0.31 ± 0.22	37.54 ± 8.86
S1-Fibre	Min	27.05	0.34	0.00	3.70	0.00	0.06	0.00	22.05
	Max	44.24	2.92	1.11	32.58	2.12	2.56	0.71	64.87
	Mean (n = 3)	41.12 ± 1.88	0.78 ± 0.20	0.13 ± 0.20	27.92 ± 2.66	0.03 ± 0.07	0.28 ± 0.36	0.05 ± 0.04	29.68 ± 4.96
S2-Fibre	Min	39.58	0.54	0.02	25.69	0	0.03	0	21.85
	Max	44.02	1.05	0.48	31.93	0.16	0.91	0.09	33.64

### TEM-EDS analysis

The mineral composition of the fibre from each AFB was determined by calculating the fibre stoichiometry, based on the EDS analytical data from Leake et al., 1997 [24]. The AFB fibre compositions reported here are also consistent with those reported by Nakamura et al., 2009 [25]. Table 1 shows that all fibres from the AFBs of non-smoking patient N1 and smoking patient S1, and one fibre from non-smoking patient N2 were amosite. Three fibres of non-smoking patient N2 and all fibres of smoking patient S2 were crocidolite. The stoichiometry of the asbestos fibres from all patients was compared with asbestos fibres from the literature [26–31]. The fibre of AFB 2 from smoking patient S1 was not evaluated due to being indistinguishable from the damage caused by EDS analysis and AFB 2 of patient S2 had no fibre.

Semi-quantitative EDS analyses for AFBs from non-smoking patient N1 (AFB only) and non-smoking patient N2 and smoking patients S1 and S2 (AFB and fibre) are shown in Table 2 and the Fe data in Fig. 4. Further plots of the EDS data can be found in the Additional file 2 (Figures S2-S16). The EDS results were normalised to give the total to be 100%.

**Fig. 4:**
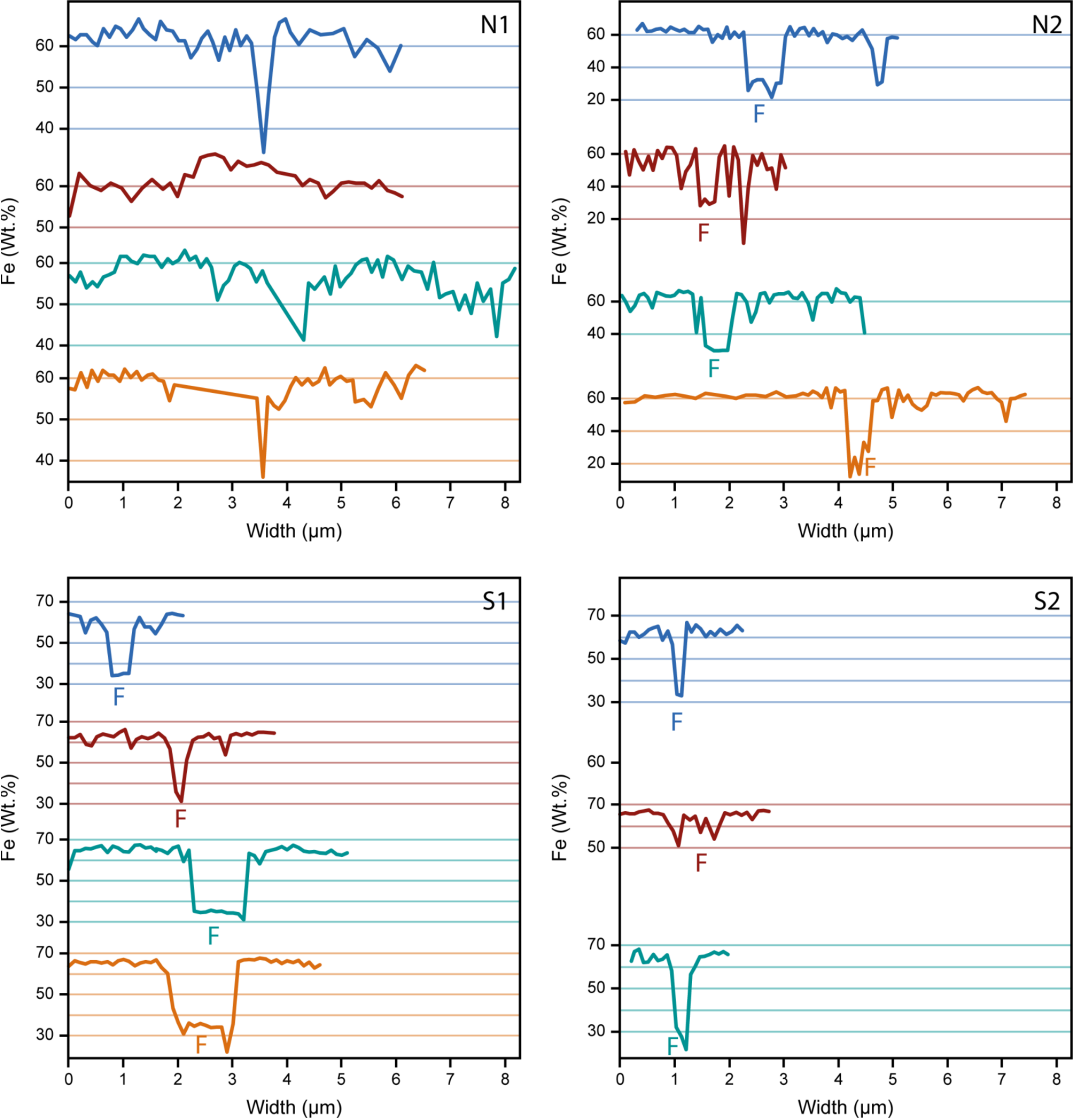
Plots of the Fe concentration from EDS against AFB size. F denotes the position of the fibre in each EDS transect. N1 and N2 are non-smoking patient 1 and 2 and S1 and S2 are smoking patient 1 and 2, respectively

**Fig. 5:**
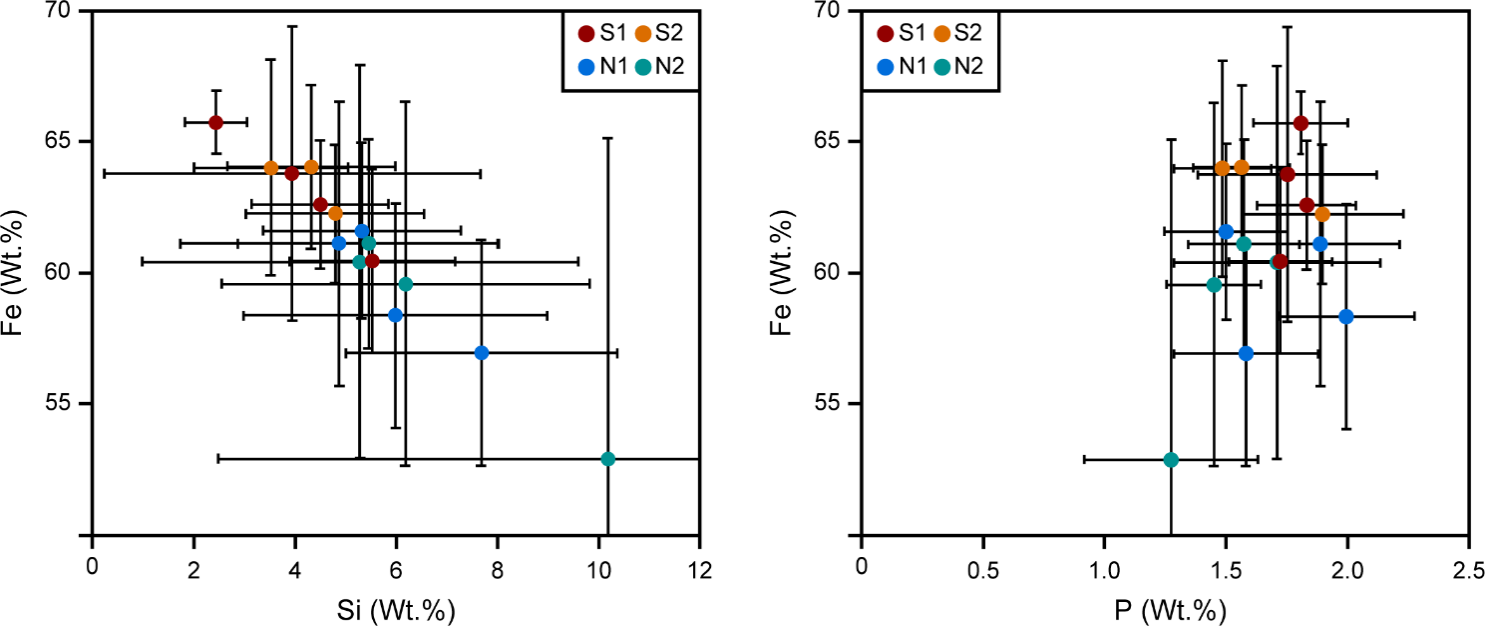
Plots of Si and P against Fe concentrations for the AFBs (excluding the fibre). N1 and N2 are non-smoking patient 1 and 2 and S1 and S2 are smoking patient 1 and 2, respectively

The results of EDS analysis show that for a given AFB the Fe, P and Ca abundances are lower in the fibre than in the rest of the AFB, while the abundance of Si is higher in the fibre than in the rest of the AFB. Furthermore, the average AFB values of Fe and Si are anti-correlated, with smokers having higher abundances of Fe and lower abundances of Si, compared to non-smokers (Fig. 5). In the non-fibre portion of the AFB, P is sometimes correlated with Fe, P often giving a low value when Fe is at low concentrations. However, this is likely due to the EDS spectrum for these points being taken where a pore space was present in the AFB and thus the overall elemental concentration was low. In terms of the average AFB values for Fe and P, there is also no correlation observed, except for the smoking patients containing higher Fe concentrations than the non-smoking patients, as mentioned previously.

### SAED patterns of the AFBs

**Fig. 6:**
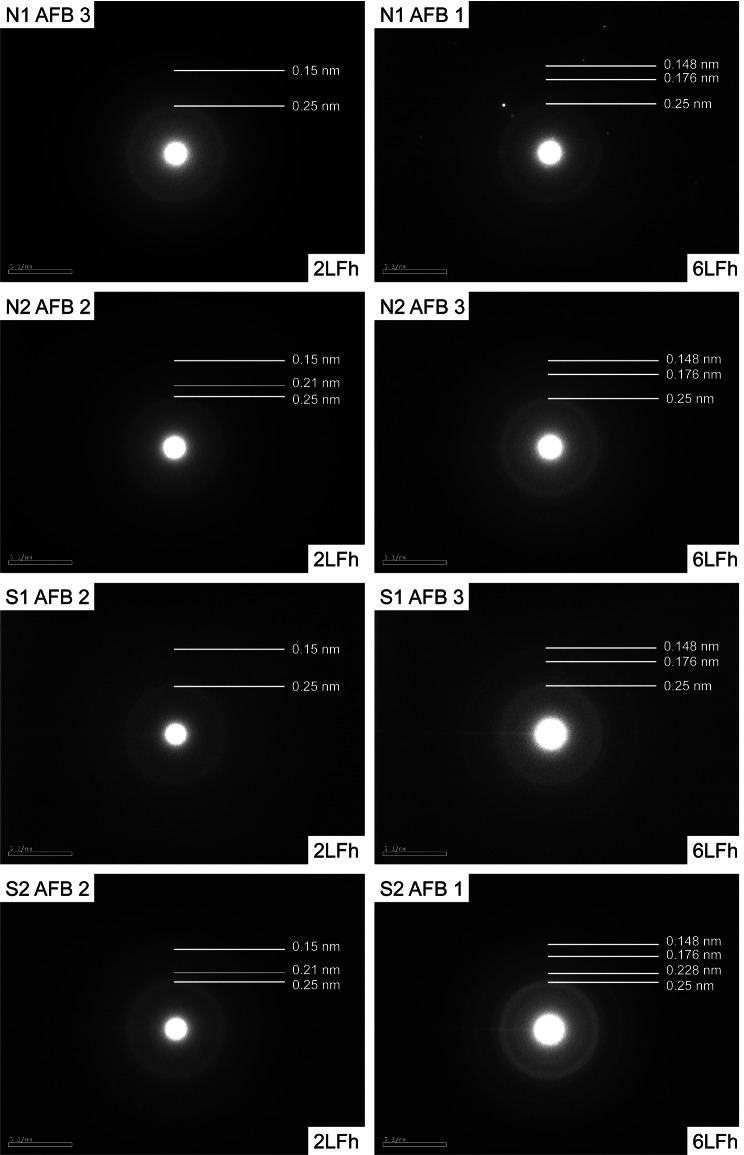
Representative SAED patterns for 2LFh and 6LFh from each patient: The white lines with numbers indicate the position of the scattering bands (thick lines) and shoulders (thin lines) in the SAED patterns. N1 and N2 are non-smoking patient 1 and 2 and S1 and S2 are smoking patient 1 and 2, respectively. Note that the 6LFh may in fact be some intermediate phase between 2LFh and 6LFh, due to the lack of 6 strong lines

SAED patterns were obtained for multiple points, including the darker and brighter layers, across each AFB of non-smoking patients N1 and N2 and smoking patients S1 and S2. Representative SAED patterns for each patient are shown in Fig. 6. Based on the number of x-ray diffraction patterns, ferrihydrite can be classified into 2 types by the number of peaks it exhibits: one small and less-ordered 2-line ferrihydrite (2LFh), and a more crystalline 6-line ferrihydrite (6LFh) [32].

All 4 patients demonstrated both the characteristic pattern of 2LFh and 6LFh, with no obvious tendency for either 2LFh or 6LFh among the smoker’s or non-smoker’s AFBs. The 2LFh featured two bright rings with d-values of ~ 0.25 nm and ~ 0.15 nm, respectively. Some SAED patterns showed shoulders on one side or both sides of each ring, such as those observed by Janney, 2000 [32]. The 6LFh did not yield 6 obvious rings, but instead demonstrated more than the 2 observed in 2LFh. As a result, it is possible that some SAED patterns interpreted as 6LFh, may in fact represent some transition phase between 6LFh and 2LFh. Nevertheless, AFB 1 of patient S2 yielded a SAED pattern with 5 clear rings and is thus likely 6LFh.

## Discussion

The external morphology of the AFBs studied here displayed a texture that suggests they were aggregates of many spherical components (e.g. Figure 1d). When the AFBs were dissected, it was apparent that some of the spherical components were distributed randomly within the internal cross-section. The spherical components were 50–170 nm in diameter, making them too small to be intact portions of macrophages. Koerten et al., 1990 [33] reported the presence of rounded iron-rich inclusions in macrophages within the peritoneal cavity of mice, which had been injected with a suspension of crocidolite fibres. The size of the spherical components identified by the current study falls within the range of the rounded iron-rich inclusions (~6 nm to >100 nm) reported by Koerten et al., 1990 [33]. Accordingly, the spherical components observed here may represent the iron-rich inclusions that formed within macrophages, and which have subsequently undergone frustrated phagocytosis.

The internal morphology of the AFBs studied here consisted of a series of accreted concentric layers rich in Fe close to the asbestos fibre of the AFBs (Fig. 2). The layers of the material deposited around the fibre of each AFB varied in terms of their brightness/darkness, which is interpreted here as relating to the density/porosity of the layers. The smoking patients’ AFBs were denser and richer in Fe than those of the non-smoking patients. Although the number of samples is limited, the smoking patients S1 and S2 presented smaller AFBs than those of the non-smoking patients. Therefore, the non-smoking patients had larger and more porous layers in their AFBs than the smoking patients. Furthermore, the mean Fe abundances for AFBs from non-smoking patients was 6.5% less than the mean Fe abundances for AFBs from the smoking patients. Such an observation is in agreement with a previous study that showed a higher Fe concentration for the dry lung tissue of smokers with AFBs compared to non-smokers with AFBs [25]. CS is known to complex Fe which is deposited in ferritin as ferrihydrite [12, 34–36]. The CS complexation of Fe, which increases Fe availability, explains the presence of the denser and more Fe-rich AFBs in the smoking patients. On the other hand, lower Fe availability in the non-smoking patients likely led to larger AFBs with more porous layers and less Fe abundance. Meanwhile, the presence of P within the AFBs is explained by the known association of ferrihydrite with phosphates [37, 38] and the presence of Ca might arise due to calcifications, which result from AM phagocytosis [21]. While Di Giuseppe et al., 2019 [11] reported Fe concentrations that were lower, and P and Si concentrations that were much higher than those reported here, the aforementioned study performed EDS analysis only on the surface of AFBs.

The SAED patterns obtained for the AFBs studied here were from either 2LFh or 6LFh [32] or possibly some intermediate ferrihydrite phase (Fig. 6). More diffuse rings were the result of a more amorphous ferrihydrite crystal while sharper rings were a result of a polycrystalline sample. The Fe in all studied AFBs was in the form of ferrihydrite and no other Fe minerals were detected, such as the goethite reported by Di Giuseppe et al., 2019 [11], which suggests that the ferritin shells were intact in the AFBs prior to sample processing. A misfolded ferritin permits exposure of ferrihydrite to the surface, leading to its phase change and its contribution to ROS production [11, 22]. However, the presence of only ferrihydrite in the AFBs studied here demonstrates that these events might not have occurred. As such, it is possible that the goethite reported previously is confined to the extremities of older AFBs and does not compose a significant proportion of the entire AFB.

Besides ROS, the onset of MM and lung cancer may be initiated by an alternate mechanism, such as inflammation [4, 5] or radiation-induced DNA damage [25]. Concerning the latter hypothesis, Ra (half-life of 1600 years) has been found in concentrations > 1 million times that of seawater in bulk analyses of AFBs. The Ra could be adsorbed onto ferrihydrite and accumulate in high concentrations in the lung environment, forming Ra hotspots that do not dissipate with time. Thus, a patient could be exposed to large doses of ionising radiation over many years.

In the smoking patients, the Fe-rich concentric layers appeared denser and the average Fe concentration of the AFBs was higher, likely due to the CS complexation of Fe, as mentioned previously [12, 34–36]. Accordingly, increased Fe mobilisation in the smoking patients could have led to denser layers, which resulted in fewer pores being incorporated into their AFBs and thus smaller AFB diameters, than non-smoking patients. Furtehrmore, the AFBs of the smoking patients showed porosity mostly around the asbestos fibres or several layers away from the asbestos fibres. However, in the non-smoking patients, there were many concentric rings visible and no consistent distribution of the porous and dense layers between AFBs, except for an initial porous layer close to the fibre, suggesting that the supply of Fe to the AFBs had varied over time and was more variable on average than that of the smokers.

**Fig. 7:**
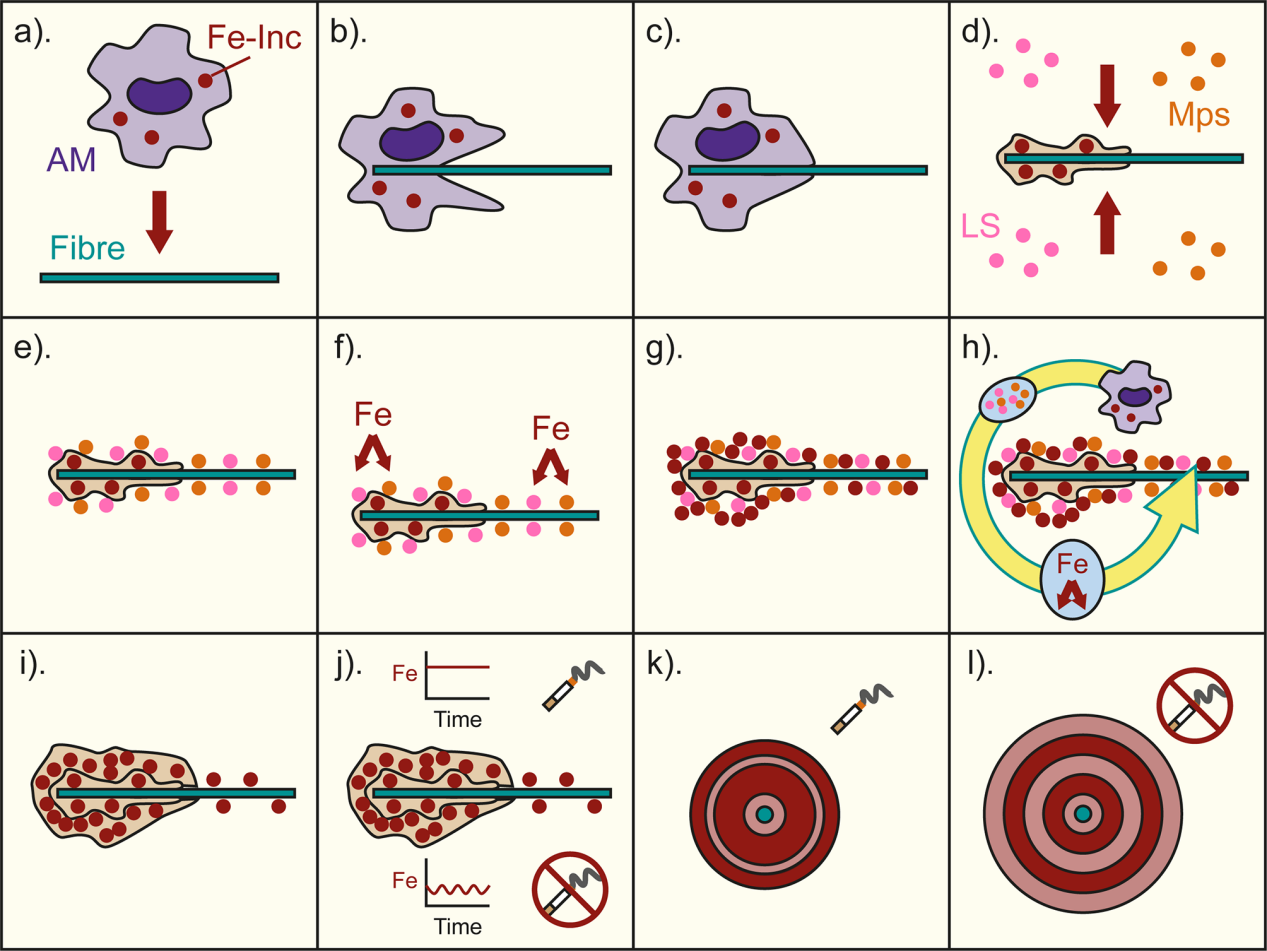
An illustration of the AFB formation model. **a**. asbestos fibres are introduced into the lung environment and AM containing Fe-rich inclusions respond as part of the body’s immune response. **b**. the AM attempt to engulf the asbestos fibre. **c**. the AM cannot fully engulf or break down the asbestos fibre and undergo frustrated phagocytosis and die. **d**. the Fe-rich inclusions and other AM material (including ferritin) are deposited on the fibre’s surface, initiating AFB formation. However, the number of Fe-rich inclusions is limited, and other sources of Fe are not yet available, resulting in an initial porous layer. Subsequently, the asbestos fibre and deposited AM material are exposed to lung surfactants (LS) and acid mucopolysaccharides (Mps). **e**. the lung surfactants and acid mucopolysaccharides coat the fibre and AM material. **f**. Fe from the lung environment is attracted to the acid mucopolysaccharides on the fibre. **g**. the Fe is adsorbed onto the surface of the asbestos fibre and AM material, initiating the formation of a dense layer surrounding the initial porous layer. **h**. repeated cycles of AM frustrated phagocytosis and Fe accumulation through adsorption result in the growth of the AFB. **i**. an example of an AFB consisting of the initial porous layer and subsequent denser layer. **j**. After continued growth, the AFBs of smokers and non-smokers differentiate due to the smoker receiving a higher and more stable supply of Fe and the non-smoker receiving a variable and on average lower supply of Fe. **k**. an example of a cross-sectional view of a smoker’s AFB, with darker red indicating a dense layer and light red a porous layer. **l**. an example of a cross-sectional view of a non-smoker’s AFB, with dark red indicating a dense layer and light red a porous layer

After consideration of the literature and the findings reported here, the following accretion model for AFB formation is proposed (Fig. 7). In the lung environment, the inhaled asbestos fibres are identified as foreign pathogens, so the AM attempt to phagocytose them (Fig. 7a-b). The AM are unable to initiate phagocytosis on the asbestos fibres due to their size and composition, leading to frustrated phagocytosis [2, 4] (Fig. 7c). The AM die on the asbestos fibres and release their components due to losing membrane integrity. The components include sequestered ferritin with scavenged Fe (possibly Fe inclusions [33]), which are deposited onto the asbestos fibre surface [4–6, 33]. While persisting in the lung environment, the asbestos fibres are coated by lung surfactant proteins and phospholipids, and acid mucopolysaccharides [1, 6, 10, 13–18] (Fig. 7d-e). The acid mucopolysaccharides having affinity to Fe, mobilise and accrete Fe and ferritin on the fibre surface (Fig. 7f-g), eventually masking the fibre’s irritant factors and ceasing its membrane damage [17, 20, 21]. As a result of the rapid initial immune response to the initial growth of the AFBs, porous layers form immediately around fibres. The exact size of the initial porous layer likely depends on the damage that the fibre inflicted on the lung tissue, as well as the specific characteristics of a given patient’s immune system, that dictate the severity of the initial immune response.

In non-smoking patients, after the initial porous layers, the asbestos fibres are masked by accretion of AM ferritin, with scavenged Fe, and lung surfactant components, including acid mucopolysaccharides. The acid mucopolysaccharides act to increase Fe accretion from the surrounding lung environment. In conjunction, the accreted AM ferritin and acid mucopolysaccharides result in subsequent dense layers after the initial porous layers. The accretion of the dense layers acts to mask the fibre from the body’s immune system and slows the immune response. Nevertheless, the presence of the AFB still attracts the attention of AM that, along with the other Fe-rich lung components, continue the growth of the AFB (Fig. 7h-i). Whether a subsequently accreted layer is dense or porous depends on the concentration of Fe in the lung at that time (Fig. 7j). Additionally, porous layers could also relate to the movement of the fibre within the lung and resulting damage to the lung tissue, which would initiate an immune response.

Meanwhile, in smoking patients Fe complexation by CS leads to a more pronounced Fe mobilisation [12, 34–36, 39] and thus a continuous supply of Fe to the AFBs. Accordingly, smoking patients experience the formation of larger denser layers around their asbestos fibres and throughout their AFBs, resulting in smaller AFB diameters (Fig. 7k). Whereas, for the non-smoking patients (Fig. 7l) the presence of more porous layers results in much larger AFBs than for the smoking patients.

The findings reported here suggest that AFB development in smoking and non-smoking patients is different. The implications of such differences on the initiation of cancer and MM, in particular, are not yet understood. However, if AFBs are denser on average in smoking patients than in non-smoking patients, then a greater amount of Fe present could result in higher levels of ROS production and thus increase the risk of developing cancer. On the other hand, unlike previous studies, all Fe was found as ferrihydrite, which infers that ferritin was likely not present as misfolded proteins. As such, the availability of exposed Fe, capable of initiating ROS formation, should have been limited during AFB formation. Therefore, other mechanisms for the onset of MM, such as inflammation or radiation-induced DNA damage, may be more likely than DNA damage from ROS.

In the radiation-induced DNA damage [25] hypothesis, denser layers of ferrihydrite could yield a larger surface area of ferrihydrite for Ra to adsorb onto and increase the overall radiation dose for smoking patients, compared to non-smoking patients. While there is no evidence that smoking increases the risk of developing asbestos-related MM, smoking has been found to have a multiplicative effect in terms of the risk of developing lung cancer in patients who have been exposed to asbestos. As such the differences in the morphology of AFBs between smoking and non-smoking patients could affect the onset of lung cancer and it may be the case that MM could be influenced in some as yet unknown manner, e.g. a shorter latency period, which should be investigated by future studies.

## Conclusions

In conclusion, multiple AFBs from two smoking and non-smoking patients were investigated in terms of their external and internal morphology. Whilst the external morphological characteristics were similar to previous studies, the internal morphology revealed differences between the AFBs of smokers and non-smokers. The region closest to the fibre of the AFBs of smokers and non-smokers was found to be porous, with a lower Fe abundance. However, in the case of smokers, the subsequent layers were denser and higher in Fe than for non-smokers.

Here it is suggested that an initial immune response likely led to an immediate porous layer, associated with lung components poor in Fe. Subsequently, ferritin and Fe-rich inclusions, which had formed in macrophages as a result of asbestos inhalation, were deposited on the fibres during frustrated phagocytosis. For smokers, after the initial immune response, the fibres were mostly masked from the body’s immune system, leading to a steady accretion of Fe, as a result of complexing by cigarette smoke and mucopolysaccharides. Nevertheless, the presence of the Fe-rich inclusions, indicates that the fibres were never fully masked and frustrated phagocytosis also continued to supply Fe to the AFBs. In the case of non-smokers, the concentration/availability of Fe was not constant but instead fluctuated over time, leading to a series of dense and porous layers, the result being overall larger and less Fe-rich AFBs.

Furthermore, SAED data indicated that the internal portions of AFBs were composed of 2LFh and 6LFh and no other Fe phases were observed, as was the case in previous studies that probed the external portions of AFBs. Such a finding may indicate that the ferritin shell was intact before sample processing and the protein was not misfolded. An intact ferritin shell would likely prevent Fe from forming ROS and so another mechanism for the onset of MM may be more likely, such as DNA damage from ionising radiation due to adsorbed Ra.

Accordingly, a higher Fe content within the AFBs of smokers may yield a higher accumulation of Ra and thus a higher radiation dose for smoking patients. Whilst no previous studies have found any links between the risk of developing asbestos-related MM and smoking, a multiplicative relationship has been observed between smoking and asbestos inhalation in the development of lung cancer. As such, smoking may have some a yet unknown influence on those who have inhaled asbestos and should be further investigated.

## Methods

### Lung samples

Human lung samples from MM patients were previously collected, digested, and quantified at Yamaguchi Ube Medical Centre for Nakamura et al., 2009 [25]. Informed consent was obtained from all patients and permission to perform research on these samples was obtained from the Institutional Review Board of the Yamaguchi Ube Medical Center. The research was carried out in accordance with the relevant guidelines. The patients’ characteristics, sample asbestos description and lung tissue digestion procedures are described in Nakamura et al., 2009 [25]. Out of the six patients from Nakamura et al., 2009, [25] patients E (N1; non-smoker), K (N2; non-smoker), H (S1; smoker) and A (S2; smoker) were selected for comparison of AFBs from a smoker vs a non-smoker perspective. Additional file 3 (Table S1) shows the characteristics of patients N1, N2, S1 and S2. Non-smoking patient N2 had 110 times fewer asbestos fibres per gram of dry lung than non-smoking patient N1 while smoking S2 had 40.5 times fewer asbestos fibres per gram of dry lung than smoking patient S1.

### SEM and FIB processing for TEM analysis

TEM analysis requires a thin sample, of under 120 nm, placed on the biofilm of a 3 mm diameter copper TEM grid. In order to achieve a thin sample, which would allow for the characterisation of the internal structure, the AFB was sliced using FIB coupled with SEM.

An aliquot of the lung sample was placed on a glass slide, left to dry overnight, and subsequently carbon-coated to ensure conductivity for SEM observations. AFBs from dried lung samples that were affixed to glass slides were selected through observations in SEM. The SEM imaging was performed using a JSM-7001F field emission-SEM (FE-SEM) and secondary electrons were employed at an accelerating voltage of 15 kV.

The AFBs identified in SEM were then placed within the JEOL JIB-4500 FIB-SEM system and sliced for TEM analysis. The accelerating voltage of the FIB was 30 kV, while that of the SEM was 20 kV. At a tilt of 0 degrees, the SEM was perpendicular to the sample, while at 52 degrees, the FIB was perpendicular to the sample. In the FIB-SEM system, the AFBs of interest for TEM analysis were located and cut at a width of less than 120 nm in order to permit the electron beam of the TEM to pass through the very thin sample. Additional file 4 (Figure S17) shows the AFB cutting procedure.

The AFB of interest was coated with gallium, for damage prevention and two trenches were dug above and below the AFB to facilitate the final lift-off of the resulting AFB slice. At an angle of 52 degrees, the first cuts of the AFB were done using a fine beam. At an angle of 0 degrees, the AFB was cut at its base. Subsequently, at an angle of 52 degrees, the final cuts of the AFB were performed using a much finer beam, to achieve a width of less than 120 nm. At the final step, the AFB extremities were cut vertically to detach the slice and permit the slice’s lift off for placement onto the biofilm, which was attached to a 3 mm diameter TEM copper grid. Lift off of the AFB slice was performed under an optical microscope by picking up the slice with a glass needle and placing the slice onto the biofilm of the TEM grid.

### TEM analysis

TEM analysis was performed with the JEM-2100F (JEOL) at an accelerating voltage of 200 kV. Pictures of the AFB slices were obtained by DF imaging, whereas the chemical composition was quantitatively obtained by EDS. The fibre crystal stoichiometry of each asbestos fibre from the AFBs was determined by EDS analysis and the formula of each asbestos fibre was calculated using the calculation procedure from Leake et al., 1997 [24].

EDS analysis was performed at a magnification of 80,000x, to better distinguish the elemental distribution within the AFBs. The large magnification of 80,000x does not allow a whole view of a given AFB. Accordingly, EDS analysis was performed from either top to bottom, or left to right, in fragmented line analyses, section by section, within the limits of the imaging program. The line analysis was composed of points spaced between 100–240 µm. Four AFBs were selected for EDS analysis from each patient N1, N2 and S1, and three AFBs from patient S2. EDS analysis data were normalised to 100%.

The crystalline nature of AFBs was obtained through SAED, represented by an array of dots or ring patterns resulting from diffracted beams of the crystalline sample.

## Electronic Supplementary Material

Below is the link to the electronic supplementary material

**Additional file 1: Figure S1:** External morphology of AFBs observed by SEM: from the non-smoking patient (a–d) N and (e–h) N2, and smoking patient (i–l) S1 and (m–o) S2, prior to cutting by FIB for TEM analysis. In each image, the area of interest is shown by a green rectangle and the Fig. number corresponds to their respective TEM image and EDS figure

**Additional file 2: Figure S2:** Internal morphology of N1:AFB1 and line profile of Fe, Si, P, and Ca. **Figure S3:** Internal morphology of N1:AFB2 and line profile of Fe, Si, P, and Ca. **Figure S4:** Internal morphology of N1:AFB3 and line profile of Fe, Si, P, and Ca. **Figure S5:** Internal morphology of N1:AFB4 and line profile of Fe, Si, P, and Ca. **Figure S6:** Internal morphology of N2:AFB1 and line profile of Fe, Si, P, and Ca. **Figure S7:** Internal morphology of N2:AFB2 and line profile of Fe, Si, P, and Ca. **Figure S8:** Internal morphology of N2:AFB3 and line profile of Fe, Si, P, and Ca. **Figure S9:** Internal morphology of N2:AFB4 and line profile of Fe, Si, P, and Ca. **Figure S10:** Internal morphology of S1:AFB1 and line profile of Fe, Si, P, and Ca. **Figure S11:** Internal morphology of S1:AFB2 and line profile of Fe, Si, P, and Ca. **Figure S12:** Internal morphology of S1:AFB3 and line profile of Fe, Si, P, and Ca. **Figure S13:** Internal morphology of S1:AFB4 and line profile of Fe, Si, P, and Ca. **Figure S14:** Internal morphology of S2:AFB1 and line profile of Fe, Si, P, and Ca. **Figure S15:** Internal morphology of S2:AFB2 and line profile of Fe, Si, P, and Ca. **Figure S16:** Internal morphology of S2:AFB3 and line profile of Fe, Si, P, and Ca

**Additional file 3: Table S1:** Mesothelioma patient details and type of asbestos present for which samples were created

**Additional file 4: Figure S17:** The sample preparation procedure to slice an AFB (S1:AFB2) for TEM analysis. (a) The positioning, (b,c) deposition, and (d,e) the trench milling of an AFB of interest. (f, g,h,i) The fine cutting, (j,k) the undercutting, and (l,m) the final side cutting of the AFB of interest

## Data Availability

The datasets generated and analysed during this study are available from the corresponding authors on reasonable request.

## Abbreviations

MM: Malignant mesothelioma
AFB: Asbestos ferruginous body
Fe: Iron
P: Phosphorus
Ca: Calcium
AM: Alveolar macrophages
ROS: Reactive oxygen species
XRF: X-ray fluorescence
SEM: Scanning electron microscopy
TEM: Transmission electron microscopy
EDS: Energy dispersive X-ray spectrometry
SAED: Selected area diffraction
FIB: Focused ion beam
CS: Cigarette smoking
N1: Non-smoking patient 1
N2: Non-smoking patient 2
S1: Smoking patient 1
S2: Smoking patient 2
DF: Dark field
Si: Silicon
2LFh: 2-line ferrihydrite
6LFh: 6-line ferrihydrite
LS: lung surfactant
Mps: mucopolysaccharides
FE-SEM: Field emission scanning electron microscope
