# Supplementary material for: An investigation of the internal morphology of asbestos ferruginous bodies: constraining their role in the onset of malignant mesothelioma
Source: Part Fibre Toxicol. 2023 May 8;20:19. doi: 10.1186/s12989-023-00522-0 (PMC10165766; doi:10.1186/s12989-023-00522-0)
Supplement: Supplementary file 1 — Additional file 1: Figure S1: External morphology of AFBs observed by SEM: from the non-smoking patient (a–d) N and (e–h) N2, and smoking patient (i–l) S1 and (m–o) S2, prior to cutting by FIB for TEM analysis. In each image, the area of interest is shown by a green rectangle and the Fig. number corresponds to their respective TEM image and EDS figure [file 12989_2023_522_MOESM1_ESM.pdf]

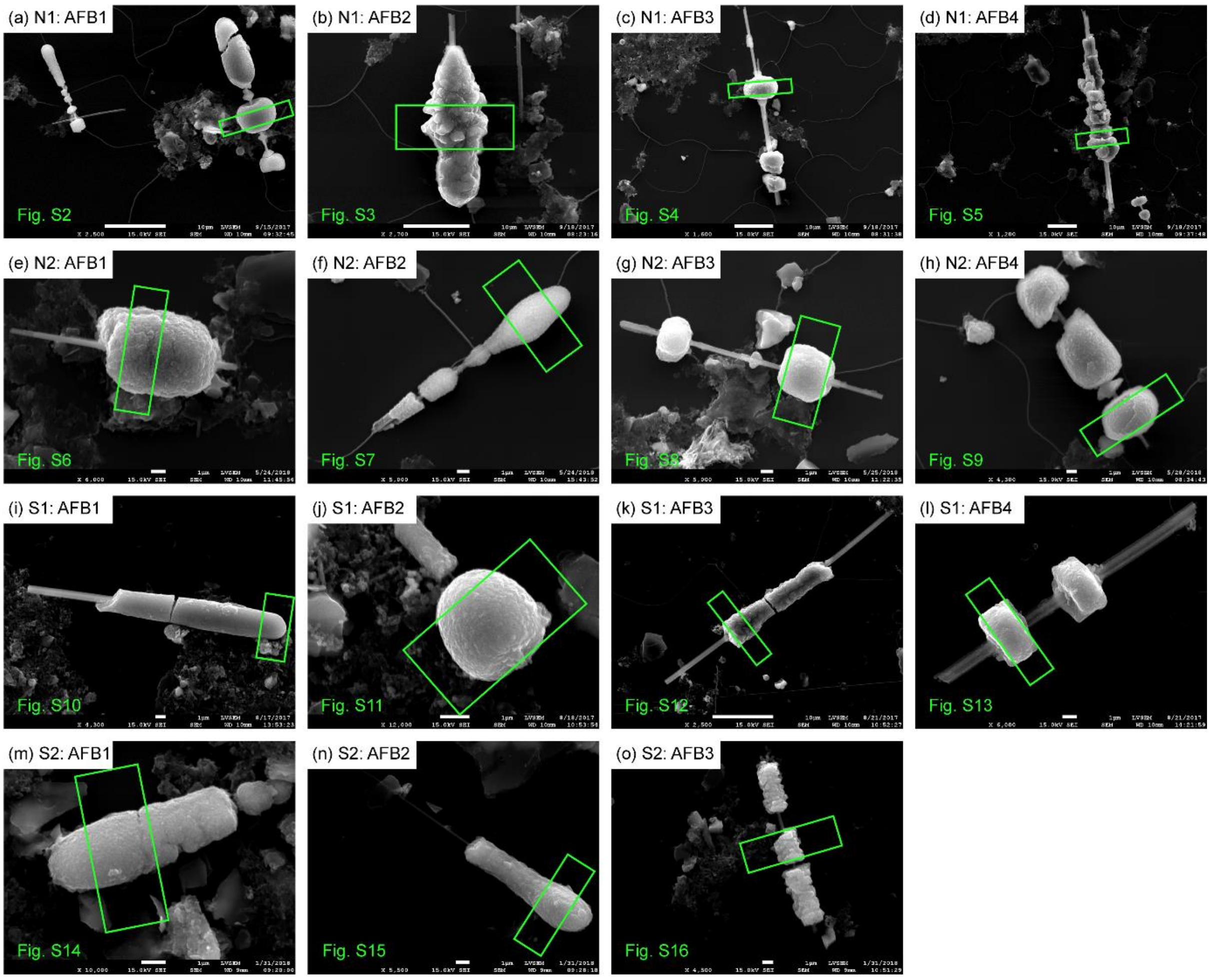

Figure S1: External morphology of AFBs observed by SEM: from the non-smoking patient (a–d) N and (e–h) N2, and smoking patient (i–l) S1 and (m–o) S2, prior to cutting by FIB for TEM analysis. In each image, the area of interest is shown by a green rectangle and the Fig. number corresponds to their respective TEM image and EDS figure.
