# Supplementary material for: An investigation of the internal morphology of asbestos ferruginous bodies: constraining their role in the onset of malignant mesothelioma
Source: Part Fibre Toxicol. 2023 May 8;20:19. doi: 10.1186/s12989-023-00522-0 (PMC10165766; doi:10.1186/s12989-023-00522-0)
Supplement: Supplementary file 2 — Additional file 2: Figure S2: Internal morphology of N1:AFB1 and line profile of Fe, Si, P, and Ca. Figure S3: Internal morphology of N1:AFB2 and line profile of Fe, Si, P, and Ca. Figure S4: Internal morphology of N1:AFB3 and line profile of Fe, Si, P, and Ca. Figure S5: Internal morphology of N1:AFB4 and line profile of Fe, Si, P, and Ca. Figure S6: Internal morphology of N2:AFB1 and line profile of Fe, Si, P, and Ca. Figure S7: Internal morphology of N2:AFB2 and line profile of Fe, Si, P, and Ca. Figure S8: Internal morphology of N2:AFB3 and line profile of Fe, Si, P, and Ca. Figure S9: Internal morphology of N2:AFB4 and line profile of Fe, Si, P, and Ca. Figure S10: Internal morphology of S1:AFB1 and line profile of Fe, Si, P, and Ca. Figure S11: Internal morphology of S1:AFB2 and line profile of Fe, Si, P, and Ca. Figure S12: Internal morphology of S1:AFB3 and line profile of Fe, Si, P, and Ca. Figure S13: Internal morphology of S1:AFB4 and line profile of Fe, Si, P, and Ca. Figure S14: Internal morphology of S2:AFB1 and line profile of Fe, Si, P, and Ca. Figure S15: Internal morphology of S2:AFB2 and line profile of Fe, Si, P, and Ca. Figure S16: Internal morphology of S2:AFB3 and line profile of Fe, Si, P, and Ca [file 12989_2023_522_MOESM2_ESM.pdf]

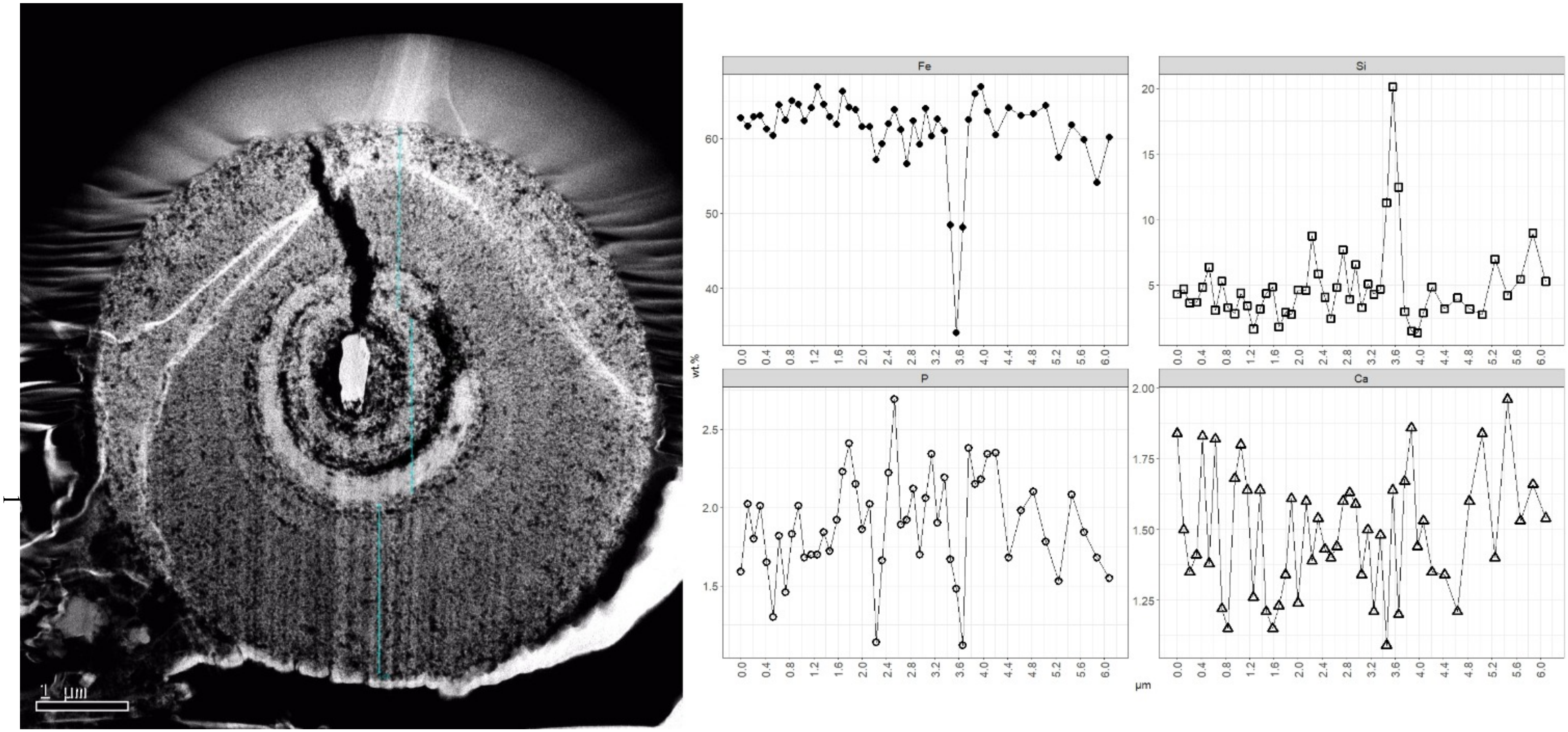

Figure S2: Internal morphology of N1:AFB1 and line profile of Fe, Si, P, and Ca.

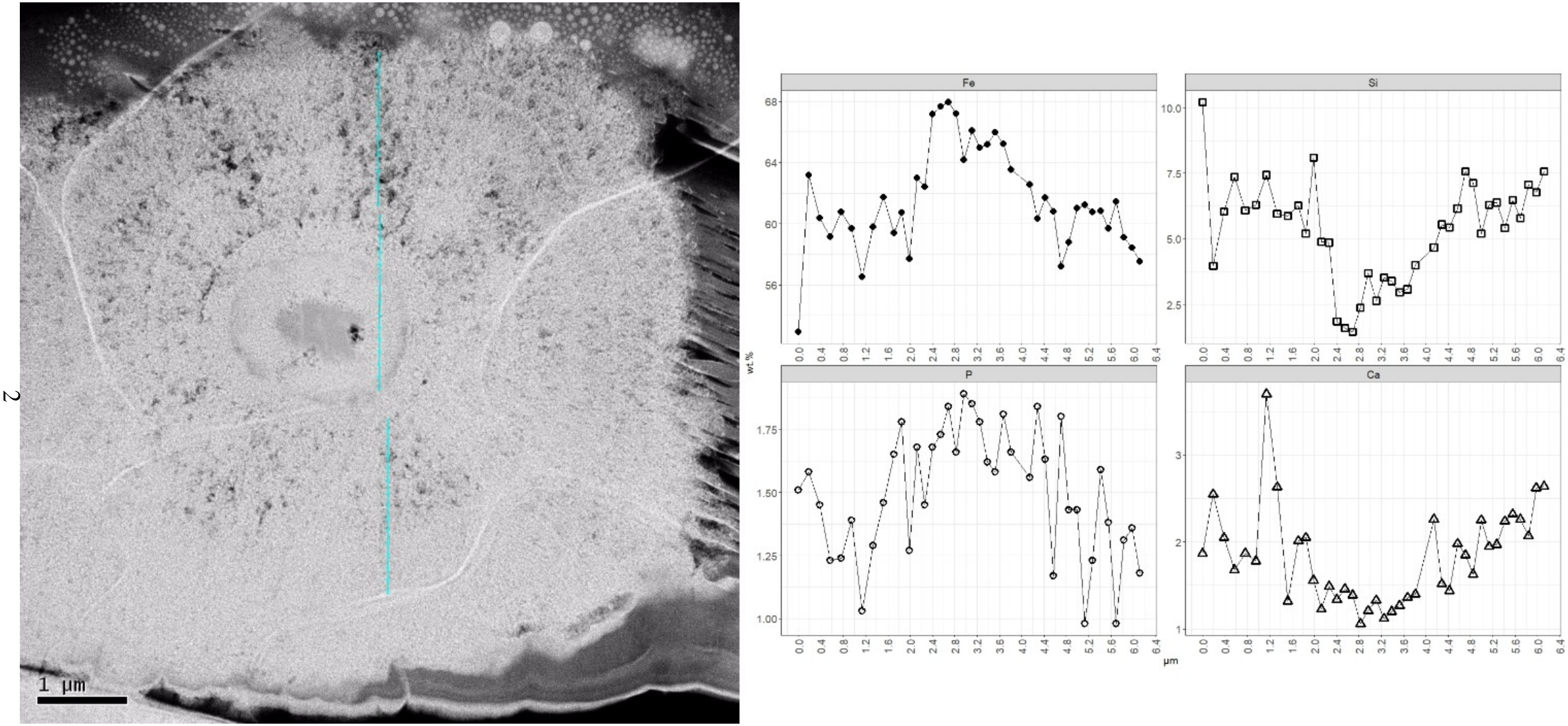

Figure S3: Internal morphology of N1:AFB2 and line profile of Fe, Si, P, and Ca.

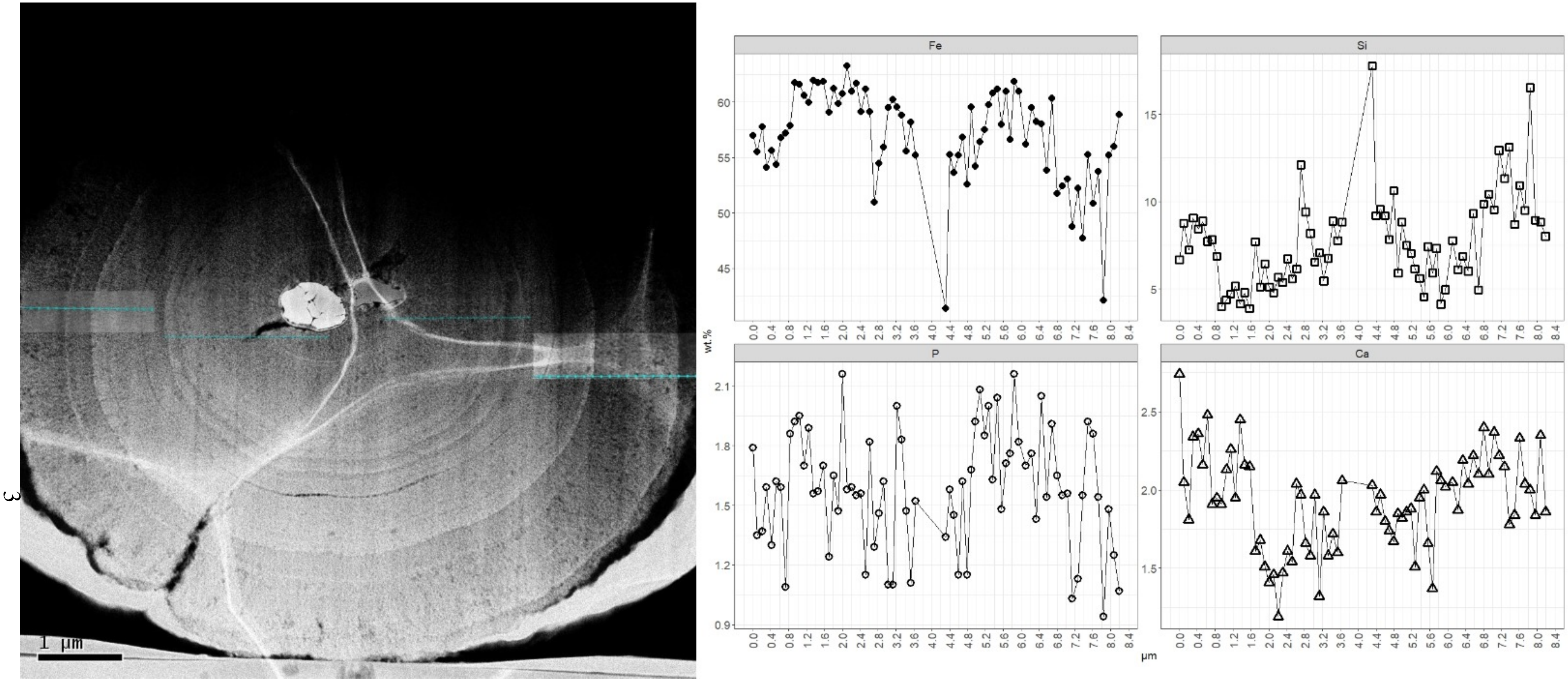

Figure S4: Internal morphology of N1:AFB3 and line profile of Fe, Si, P, and Ca.

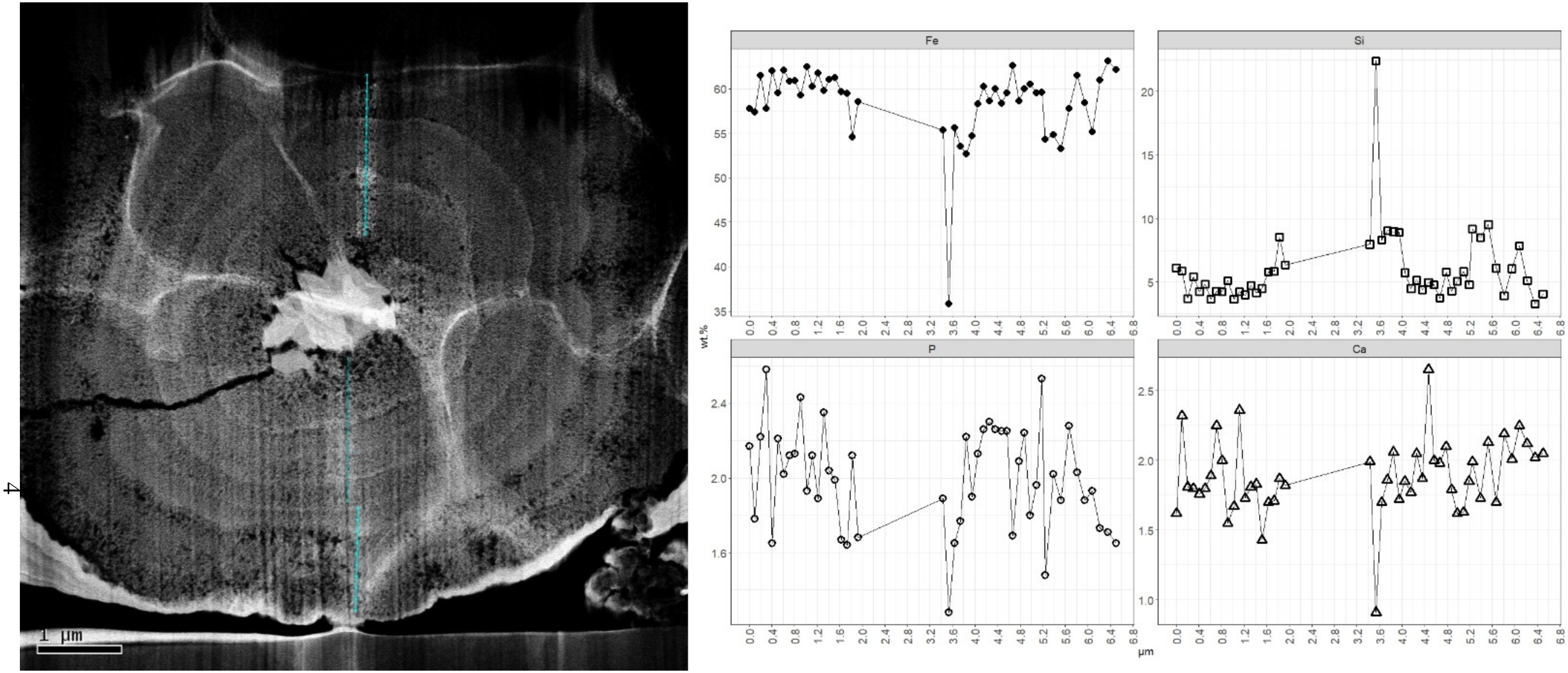

Figure S5: Internal morphology of N1:AFB4 and line profile of Fe, Si, P, and Ca.

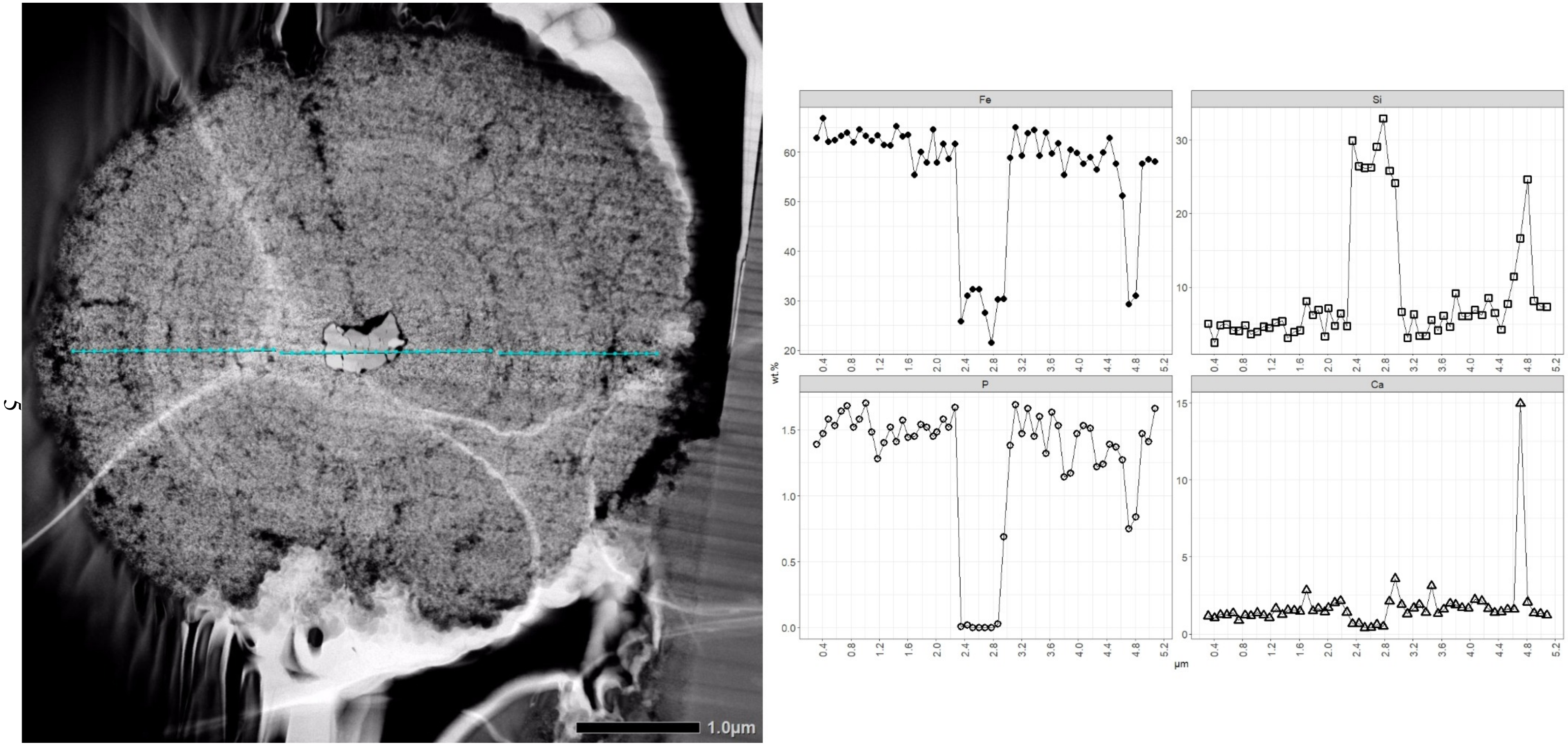

Figure S6: Internal morphology of N2:AFB1 and line profile of Fe, Si, P, and Ca.

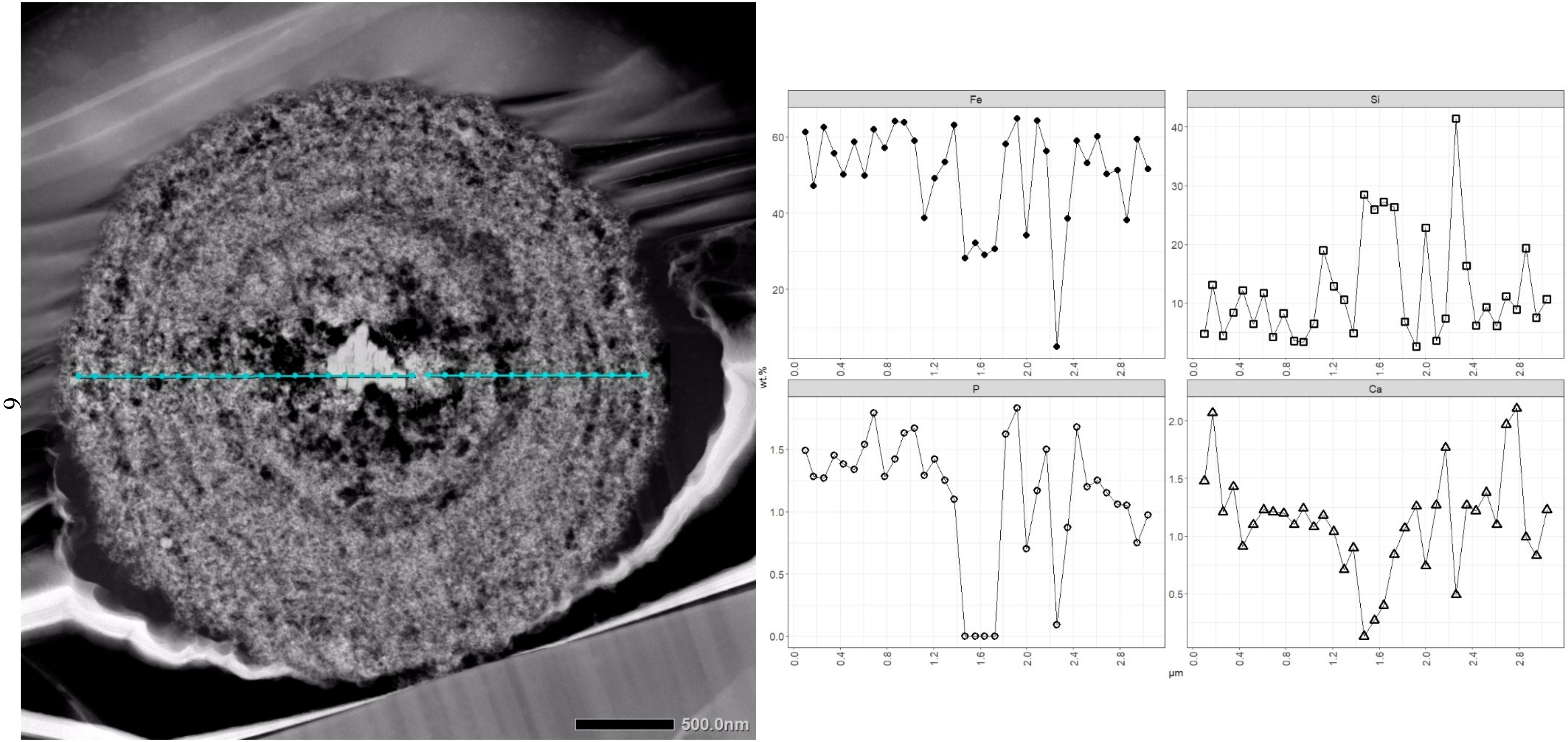

Figure S7: Internal morphology of N2:AFB2 and line profile of Fe, Si, P, and Ca.

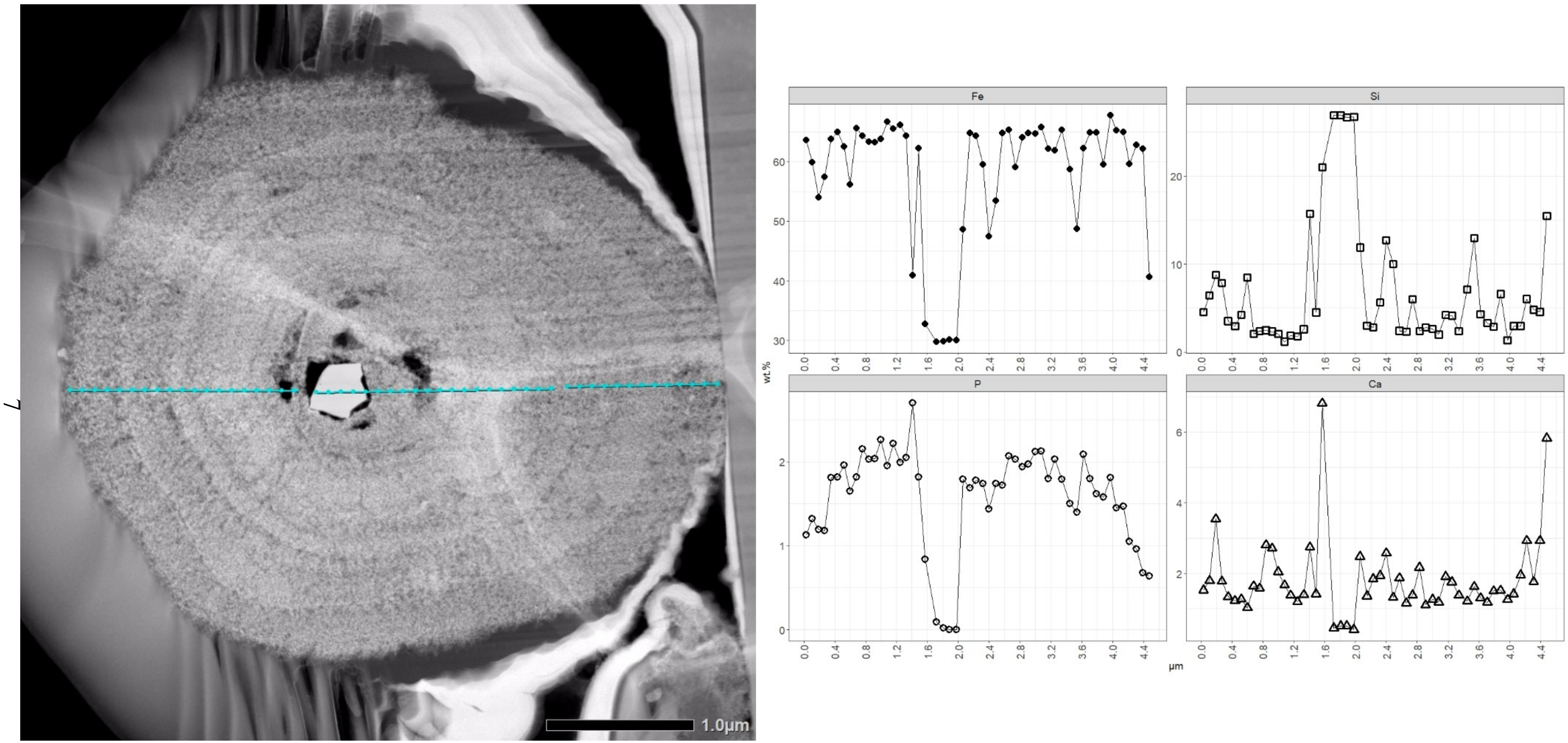

Figure S8: Internal morphology of N2:AFB3 and line profile of Fe, Si, P, and Ca.

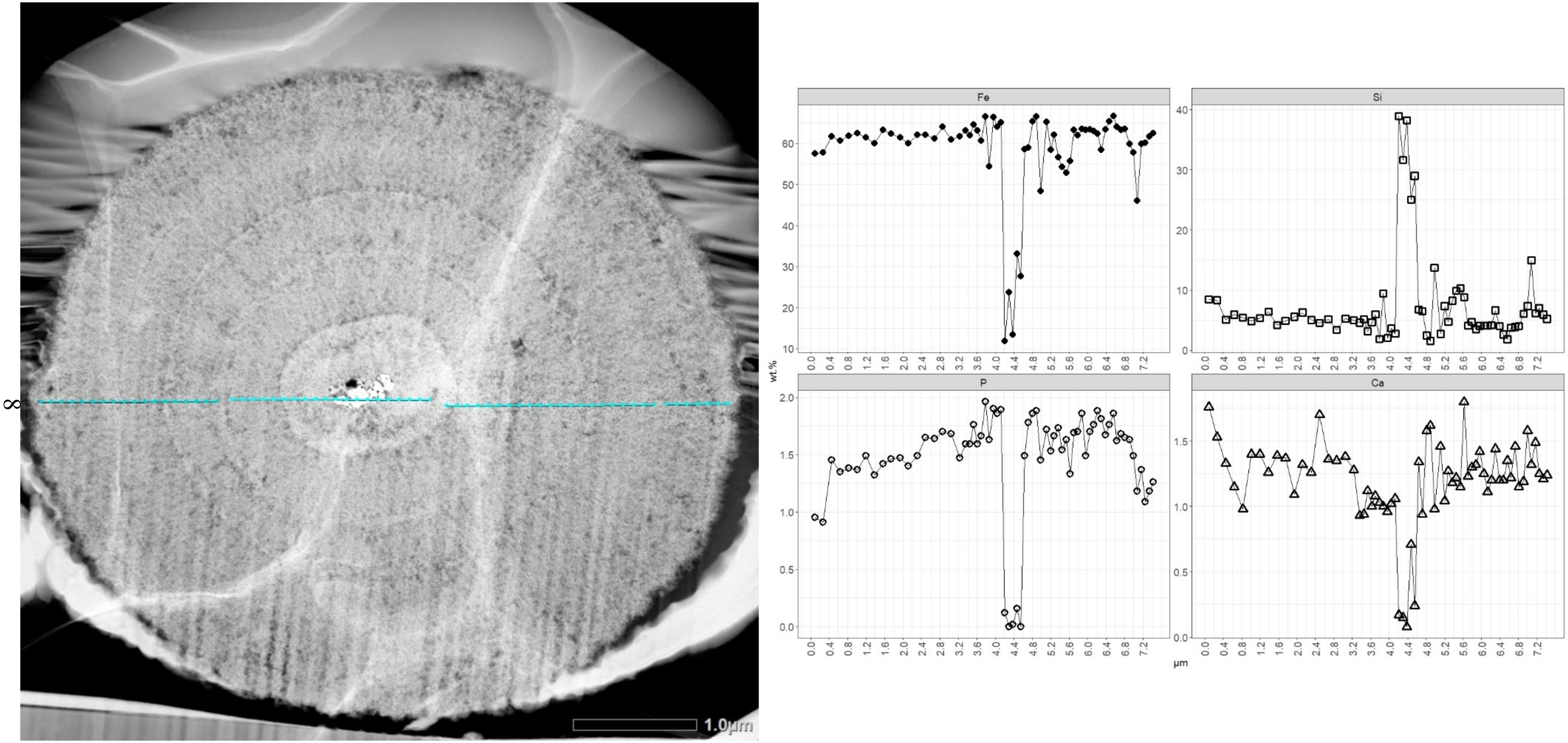

Figure S9: Internal morphology of N2:AFB4 and line profile of Fe, Si, P, and Ca.

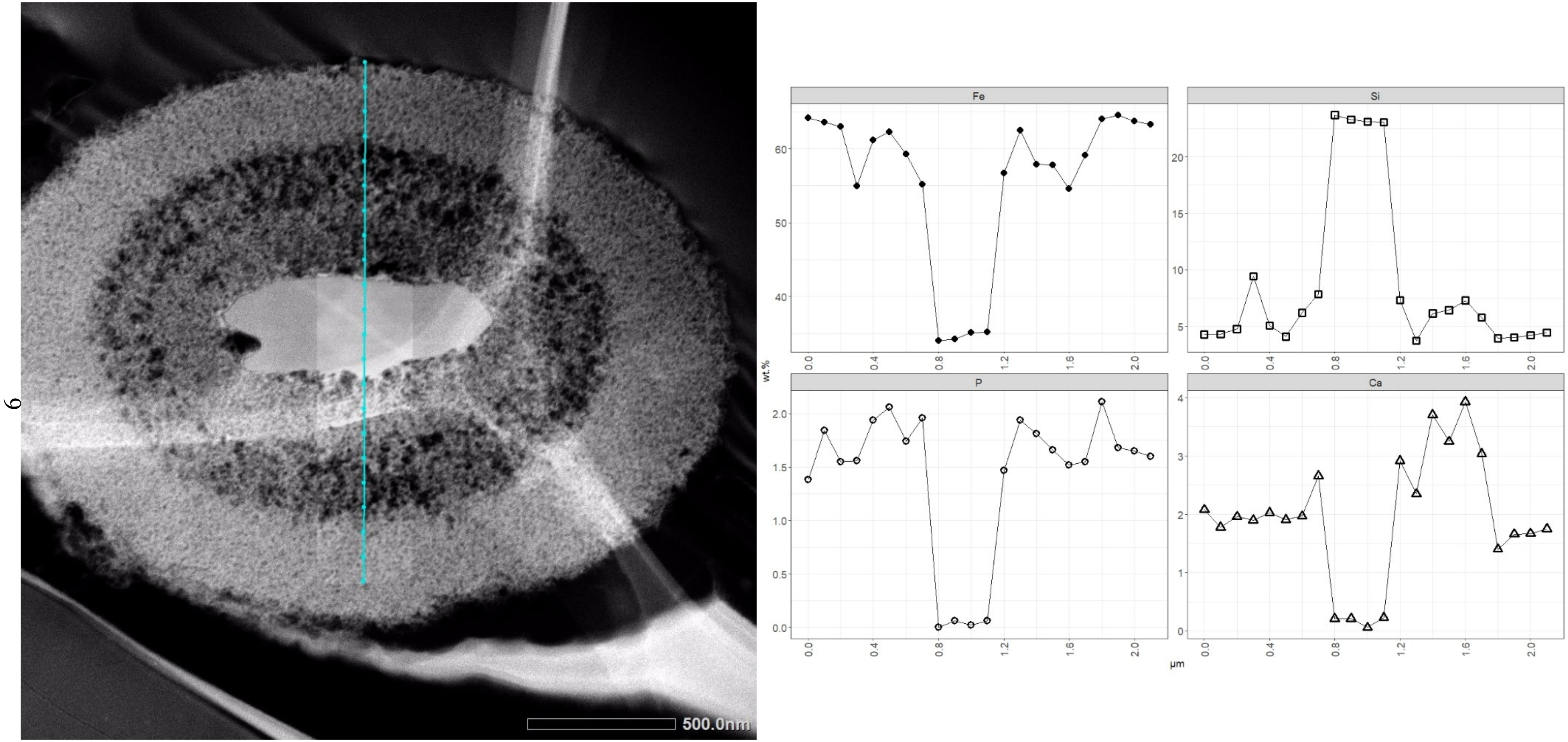

Figure S10: Internal morphology of S1:AFB1 and line profile of Fe, Si, P, and Ca.

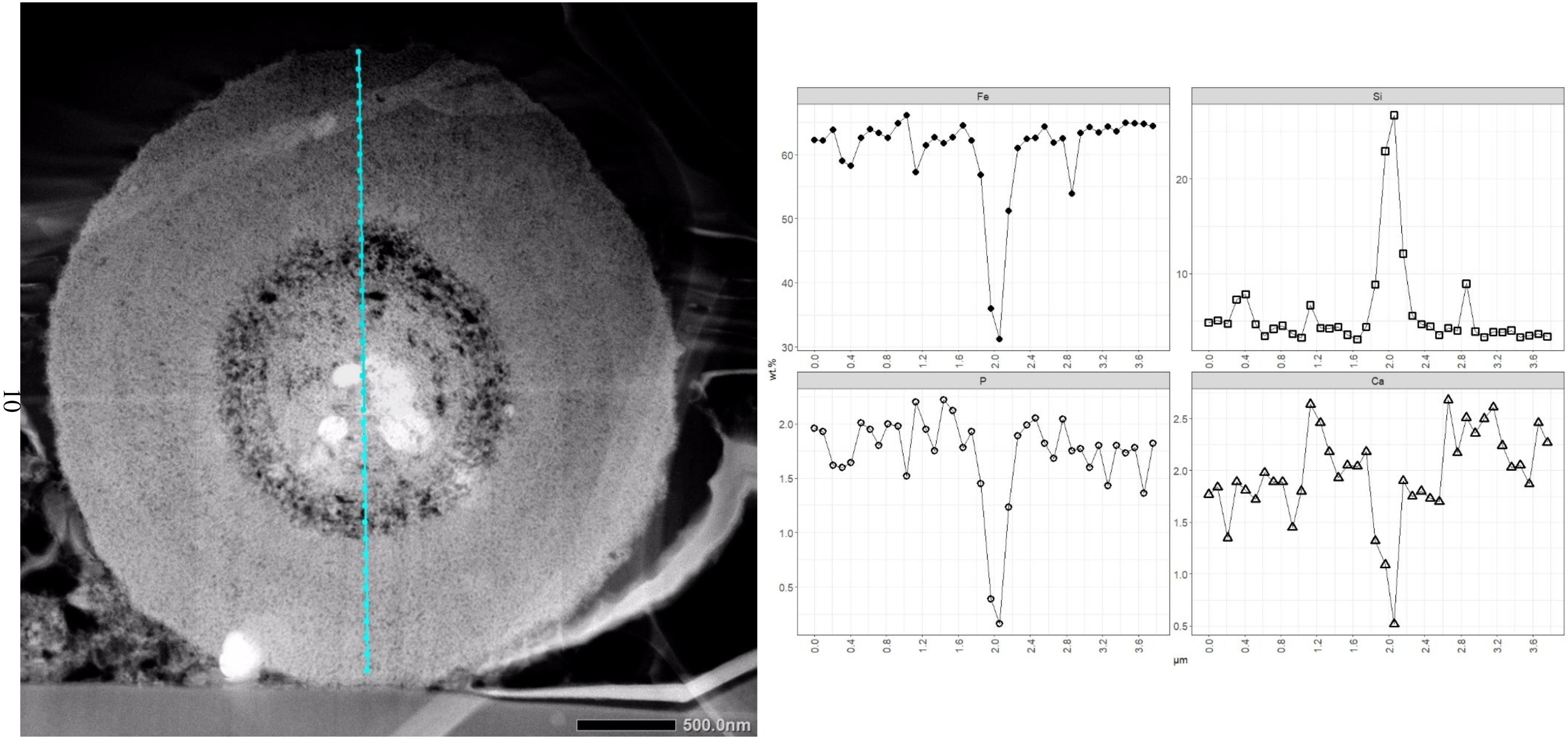

Figure S11: Internal morphology of S1:AFB2 and line profile of Fe, Si, P, and Ca.

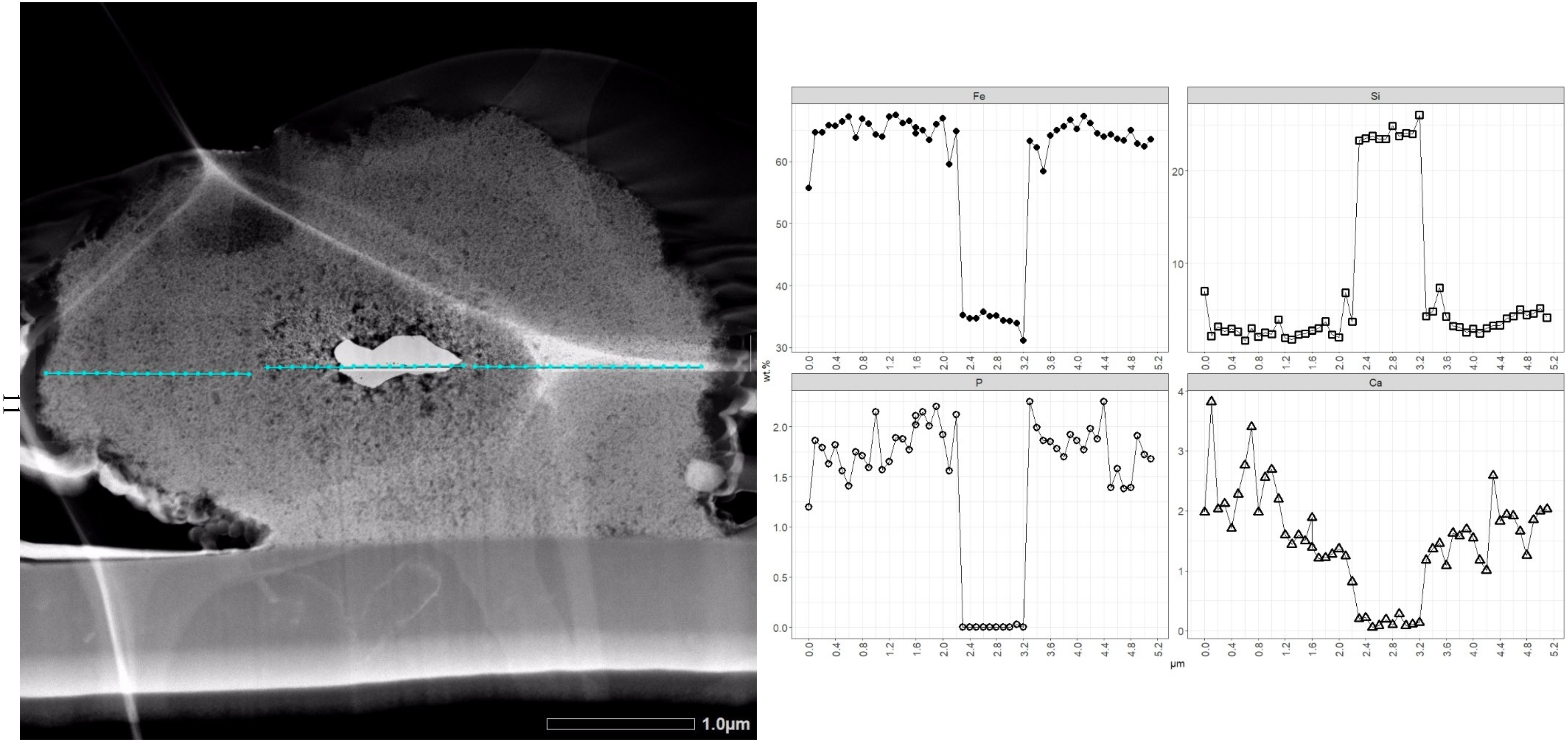

Figure S12: Internal morphology of S1:AFB3 and line profile of Fe, Si, P, and Ca.

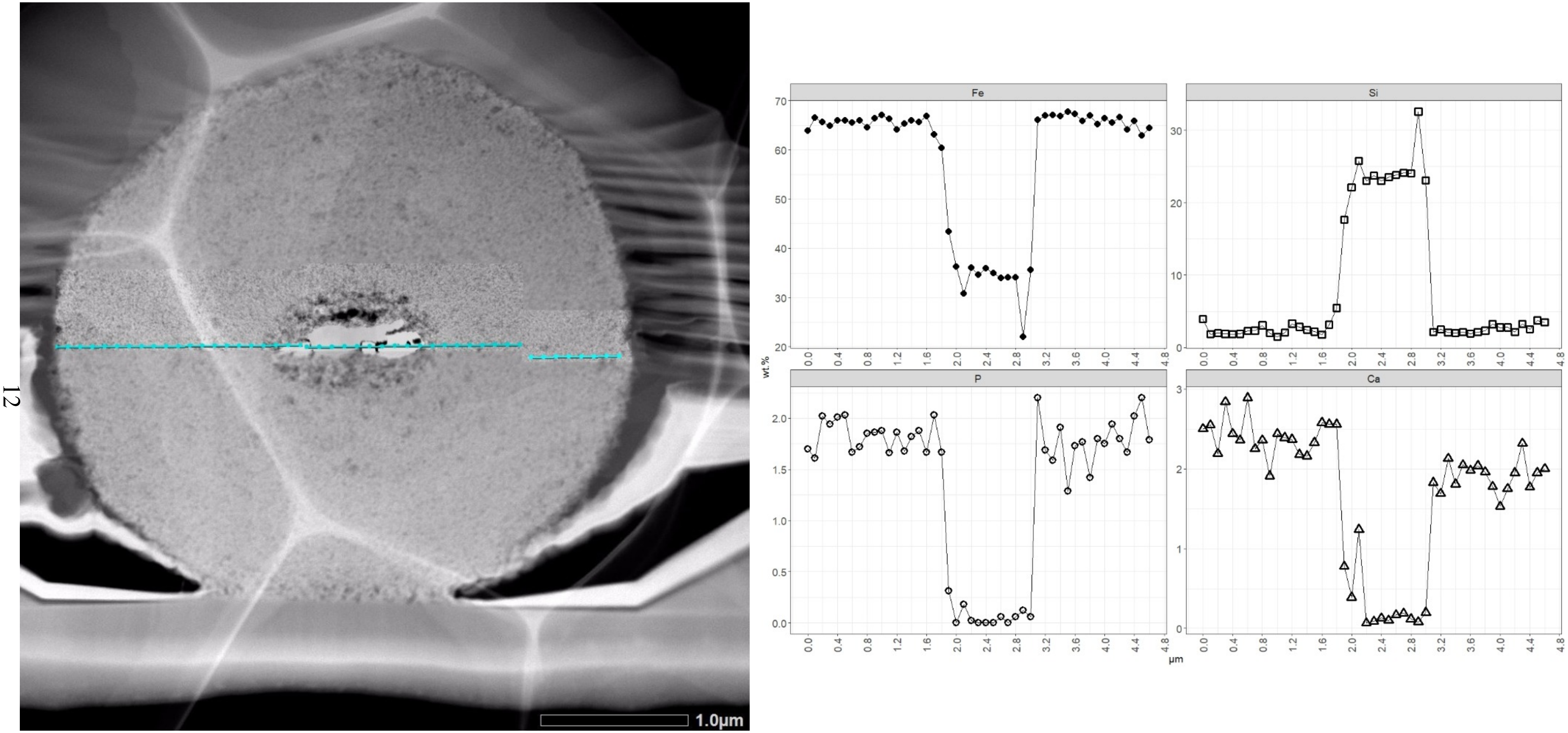

Figure S13: Internal morphology of S1:AFB4 and line profile of Fe, Si, P, and Ca.

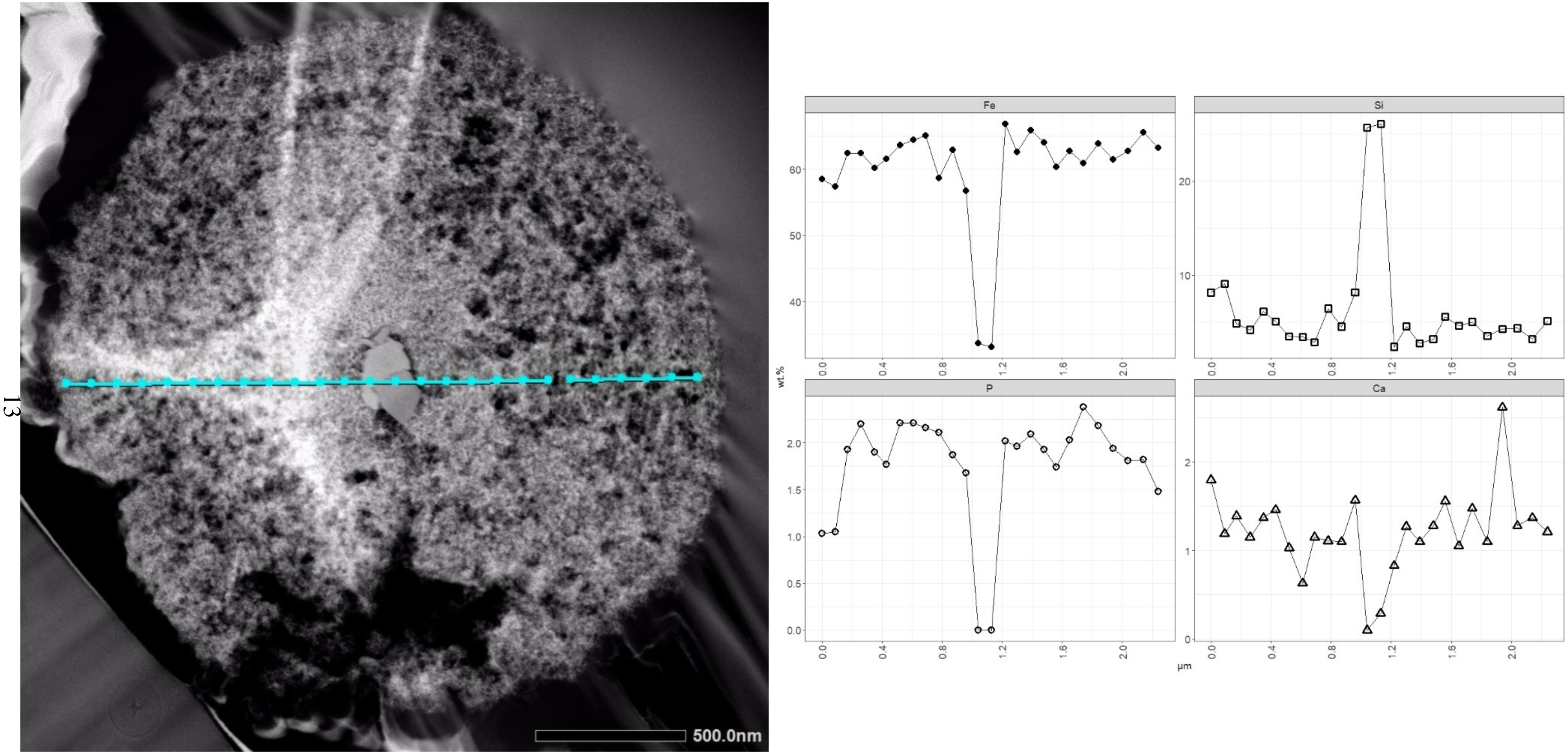

Figure S14: Internal morphology of S2:AFB1 and line profile of Fe, Si, P, and Ca.

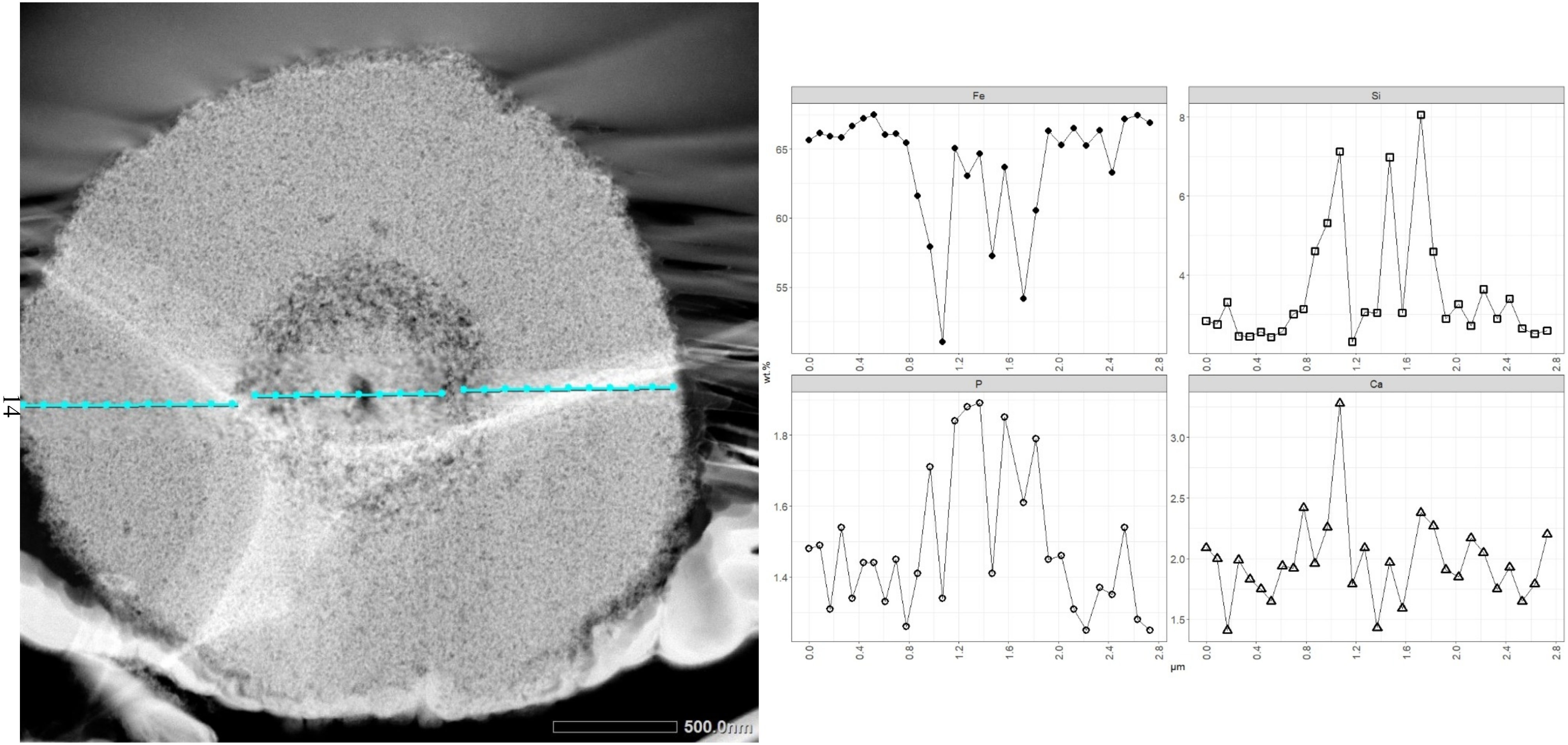

Figure S15: Internal morphology of S2:AFB2 and line profile of Fe, Si, P, and Ca.

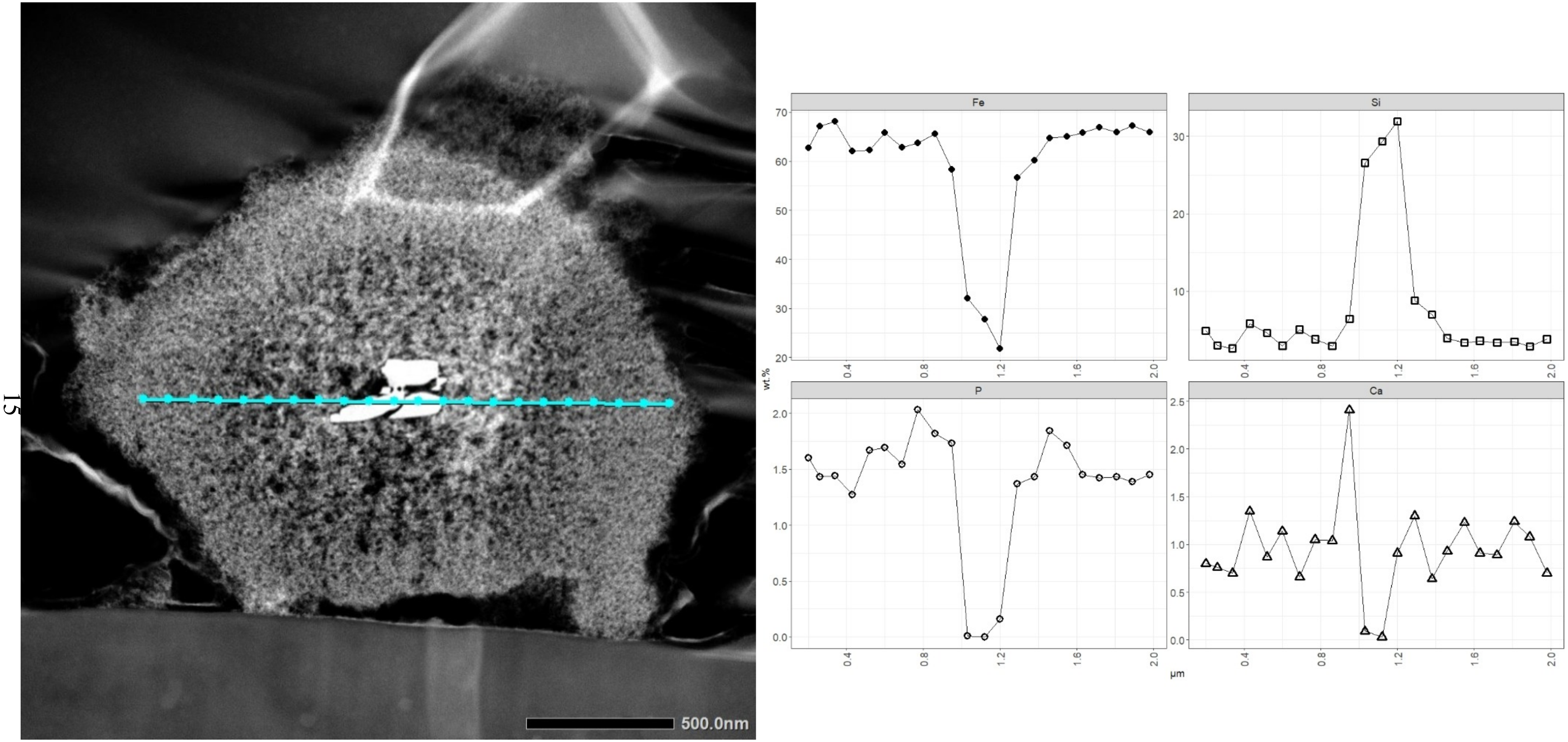

Figure S16: Internal morphology of S2:AFB3 and line profile of Fe, Si, P, and Ca.
