# Supplementary material for: An investigation of the internal morphology of asbestos ferruginous bodies: constraining their role in the onset of malignant mesothelioma
Source: Part Fibre Toxicol. 2023 May 8;20:19. doi: 10.1186/s12989-023-00522-0 (PMC10165766; doi:10.1186/s12989-023-00522-0)
Supplement: Supplementary file 3 — Additional file 3: Table S1: Mesothelioma patient details and type of asbestos present for which samples were created [file 12989_2023_522_MOESM3_ESM.pdf]

Table S1: Mesothelioma patient details and type of asbestos present for which samples were created.

patient	N1 (non smoking)	N2 (non smoking)	S1 (smoking)	S2 (smoking)
age	65	51	63	65
gender	F	F	M	M
type of MM	biphagic	epitheloid	biphagic	biphagic
initial symptom	chest pain	chest pain	cough	cough
occupation	insulation industry	Post office	asbestos scrapper	quarrying industry
smoking experience	none	none	40 years	35 years
			Quit 1 year before operation	Quit 6 months before operation
surgery	left extrapleural pneumonectomy	left extrapleural pneumonectomy	Autopsy	Left extrapleural pneumonectomy
surgery date	July, 2007	November, 2007	December, 2003	October, 2007
AFB per dry lung	$4.4x10^5$ fibre g^{-1}	$4.0x10^3$ fibre g^{-1}	$3.2x10^5$ fibre g^{-1}	$7.9x10^3$ fibre g^{-1}
