# Supplementary material for: An investigation of the internal morphology of asbestos ferruginous bodies: constraining their role in the onset of malignant mesothelioma
Source: Part Fibre Toxicol. 2023 May 8;20:19. doi: 10.1186/s12989-023-00522-0 (PMC10165766; doi:10.1186/s12989-023-00522-0)
Supplement: Supplementary file 4 — Additional file 4: Figure S17: The sample preparation procedure to slice an AFB (S1:AFB2) for TEM analysis. (a) The positioning, (b,c) deposition, and (d,e) the trench milling of an AFB of interest. (f, g,h,i) The fine cutting, (j,k) the undercutting, and (l,m) the final side cutting of the AFB of interest [file 12989_2023_522_MOESM4_ESM.pdf]

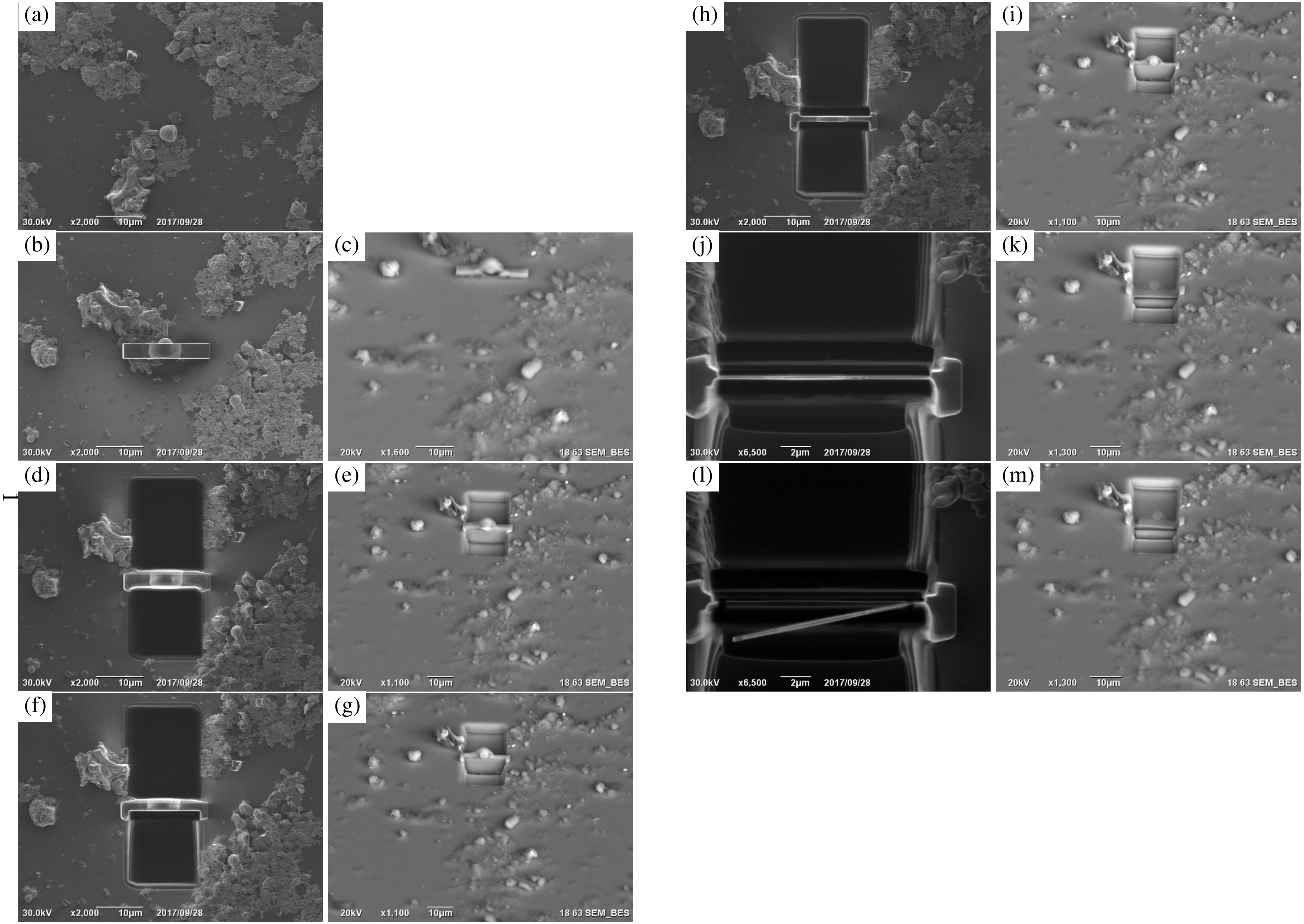

Figure S17: The sample preparation procedure to slice an AFB (S1:AFB2) for TEM analysis. (a) The positioning, (b,c) deposition, and (d,e) the trench milling of an AFB of interest. (f,g,h,i) The fine cutting, (j,k) the undercutting, and (l,m) the final side cutting of the AFB of interest.
